# Network Pharmacology Approach to Explore the Skin‐Lightening Compounds and Potential Mechanisms of Chinese Herbal Medicines

**DOI:** 10.1111/jocd.70562

**Published:** 2025-12-05

**Authors:** Yixuan Liang, Jing Chen, Chuhan Fu, Xixia Dai, Ling Jiang, Xinxin Lei

**Affiliations:** ^1^ Department of Dermatology The Third Xiangya Hospital, Central South University Changsha P.R. China; ^2^ Department of Dermatology, Hangzhou Third People’s Hospital Hangzhou Third Hospital Affiliated to Zhejiang Chinese Medical University Hangzhou P.R. China

**Keywords:** acacetin, melanogenesis, network pharmacology, quercetin, tyrosinase

## Abstract

**Introduction:**

Herbal formulations have been used for skin whitening in China for centuries, yet systematic evaluations of their efficacy and underlying mechanisms remain limited. This study aims to identify potential anti‐melanogenic compounds in traditional skin‐whitening herbs using network pharmacology and investigate their mechanisms of action.

**Methods:**

The Traditional Chinese Medicine Systems Pharmacology (TCMSP) database was used to screen for anti‐melanogenic compounds and their potential targets. A protein–protein interaction (PPI) network was constructed using Cytoscape 3.7.2, followed by KEGG pathway enrichment analysis. The pharmacological effects were assessed in a 3D human skin model, and in vitro experiments were conducted to explore the molecular mechanisms of the identified compounds.

**Results:**

Eight potential skin‐whitening herbs were identified, including *Ampelopsis japonica*, *Nepeta tenuifolia* Benth., *Scutellaria baicalensis* Georgi, *Asarum heterotropoides* F. Schmidt, *Ipomoea nil* , *Angelica dahurica*, *Angelica sinensis*, and *Prunus persica* , along with ten active compounds: quercetin, kaempferol, wogonin, beta‐sitosterol, baicalein, stigmasterol, anhydroicaritin, agroclavin, moslosooflavone, and acacetin. Among them, quercetin and acacetin demonstrated significant anti‐melanogenic effects in the 3D skin model by downregulating ET‐1, VEGF, and PTGS2 in keratinocytes. Molecular docking analysis further revealed strong binding affinities of quercetin and acacetin to TLR4, CHUK, and RELA in the NF‐κB pathway, suggesting their role in melanogenesis inhibition via suppression of keratinocyte paracrine signaling.

**Conclusions:**

This study highlights the potential of network pharmacology as an effective approach for screening anti‐melanogenic compounds from traditional skin‐whitening herbs. Quercetin and acacetin were identified as key bioactive compounds that inhibit melanogenesis by modulating keratinocyte signaling pathways.

**Abbreviations**
α‐MSH: α‐melanocyte stimulating hormone
ACTH: adrenocorticotropic hormone
BC: betweenness centrality
bFGF: basic fibroblast growth factor
CC: closeness centrality
COX‐2: cyclooxygenase‐2
DC: degree centrality
DL: drug‐likeness
ET‐1: endothelin‐1
ETRB: endothelin B receptor
GM‐CSF: granulocyte–macrophage colony‐stimulating factor
HE: hematoxylin–eosin
HQ: hydroquinone
IL: interleukin
MITF: microphthalmia‐associated transcription factor
NF‐κB: nuclear factor kappa B
OB: oral bioavailability
PG: prostaglandin
PKC: protein kinase C
PPI: protein–protein interaction
SCF: stem cell factor
TCM: traditional Chinese medicine
TCMSP: traditional Chinese medicine systems pharmacology
TNF: tumor necrosis factor
TYR: tyrosinase
TYRP: tyrosinase‐related protein
VEGF: vascular endothelial growth factor

## 1 Introduction

Melanin is synthesized by the melanosomes in melanocytes located in the basal layer of the skin, and subsequently transferred through its dendrites to the surrounding keratinocytes, eventually resulting in skin pigmentation [1]. Tyrosinase (TYR), tyrosinase‐related protein (TYRP)‐1 and TYRP2 are the key enzymes involved in melanin synthesis [2, 3], and are expressed in the melanocytes under the control of the microphthalmia‐associated transcription factor (MITF) [4, 5]. In addition, the cytokines secreted by the keratinocytes, fibroblasts, vascular endothelial cells and immune cells regulate melanin synthesis upon binding to the surface receptors on melanocytes [5, 6]. The pro‐melanogenic keratinocyte‐derived factors include basic fibroblast growth factor (bFGF), stem cell factor (SCF), granulocyte–macrophage colony‐stimulating factor (GM‐CSF), α‐melanocyte stimulating hormone (α‐MSH), adrenocorticotropic hormone (ACTH), endothelin‐1 (ET‐1), prostaglandin (PG)E2 and PGF2α, and vascular endothelial growth factor (VEGF) [7]. ET‐1 promotes melanogenesis by binding to the endothelin B receptor (ETRB) and activating the protein kinase C (PKC) pathway [8]. Likewise, VEGF promotes melanin synthesis by binding to VEGF receptors on melanocytes and activating inflammatory pathways [9, 10]. In addition, an inflammatory milieu stimulates production of cyclooxygenase‐2 (COX‐2), which catalyzes the synthesis of PGE2 [11, 12]. Both PGE2 and PGF2α induce melanogenesis by promoting the differentiation of melanocyte dendrites and upregulating TYR activity via the PLC‐protein kinase C pathway [13]. Recent studies have shown that inflammatory cytokines, such as tumor necrosis factor (TNF)‐α and interleukin (IL)‐6, also regulate melanin synthesis [6].

While melanin protects the skin from UV‐induced skin photoaging and carcinogenesis, excessive activation of melanocytes leads to hyperpigmentation, which can manifest as freckles and melasma [14, 15]. Kojic acid, hydroquinone (HQ), and vitamin C are routinely used to reverse skin hyperpigmentation [16]. Although kojic acid and HQ significantly inhibit melanogenesis, the associated adverse effects, such as skin irritation and disruption of the skin barrier, have limited their use to some extent [17, 18]. On the other hand, vitamin C can lighten skin pigmentation through antioxidant effects as well as inhibition of TYR activity, but its poor stability and permeability also limit clinical application [19]. In recent years, studies have shown that some Chinese herbs can achieve skin whitening by inhibiting melanin production. For example, Astragalus membranaceus extract can inhibit melanin synthesis through the ERK pathway [20]. In addition, the extract of adlay seeds can achieve skin whitening by antioxidant activity and via downregulation of MITF, TYR, TYRP1, and DCT [21]. The stem and leaf extracts of *Panax ginseng* have also demonstrated have anti‐TYR activity [22, 23]. Resveratrol, a polyphenol present in several herbs, inhibits melanogenesis by downregulating COX‐2 through the ERK1/2 and PI‐3K/Akt pathways [11]. In addition, polysaccharides derived from the fungus Ganoderma lucidum inhibit melanin synthesis by blocking the paracrine effects of keratinocytes through the STAT3/FGF2 pathway [14].

Herbal medicine has been used for thousands of years in China to treat various ailments. Several encyclopedias, such as *Essential Prescriptions Worth a Thousand Gold*, *Liu Juanzi's Remedies Bequeathed by Ghosts*, *Orthodox Manual of External Diseases*, *Golden Mirror of Medicine*, have recorded herbal formulas for beauty and skin care. Some of these herbal prescriptions have skin whitening effects and have been used in clinical practice. Sibai Quban Ruangao, which is recommended in the Expert Consensus on the Treatment of Melasma in Chinese Medicine [24], contains *Angelica dahurica* (Hoffm.) Benth. & Hook.f. ex Franch. & Sav. (Baizhi), *Ampelopsis japonica* (Thunb.) Makino (Bailian), and *Sauromatum giganteum* (Engl.) Cusimano & Hett. (Baifuzi). However, the anti‐melanogenic herbs and active compounds in these prescriptions have not been identified so far. Given the complexity of herbal formulations, their mechanisms of action involve multiple targets and pathways that have not been completely elucidated. In addition, the considerable gap between basic pharmacological research and clinical studies, and the batch differences in the composition of herbal medicines due to non‐standardized extraction methods also limit their clinical applications [25]. Therefore, a systematic and comprehensive screening method is urgently needed for studying traditional Chinese medicine (TCM) formulations.

**FIGURE 1:**
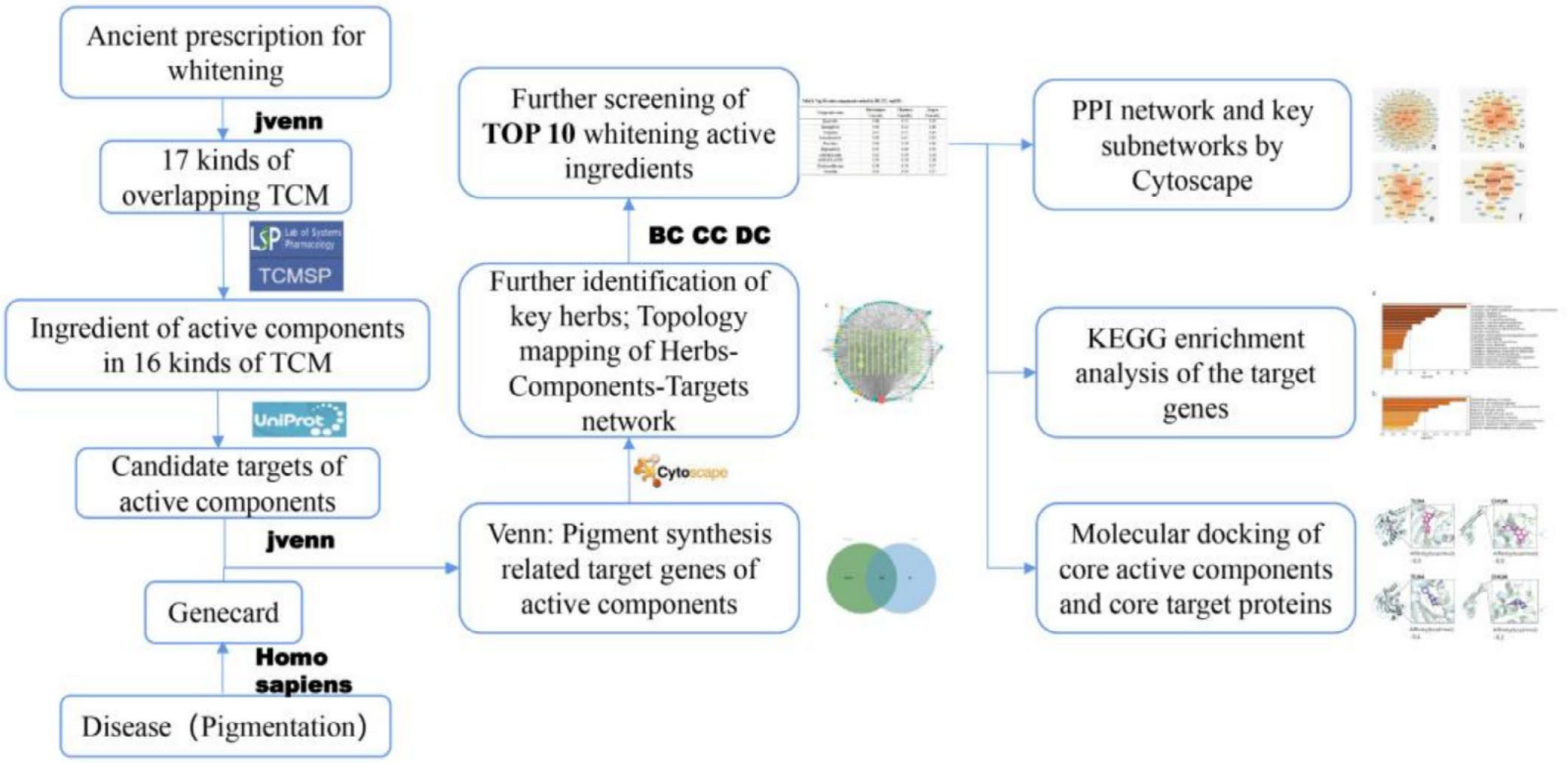
Workflow for exploring anti‐melanogenic active components and pharmacological mechanisms of TCM formulations.

Network pharmacology has proved to an effective method for identifying the active compounds of TCM formulations [26]. It is based on various biological and bioinformatics methodologies that screen active compounds, discover new drug targets, and elucidate the mechanisms of action from a holistic perspective [27, 28]. The aim of this study was to apply network pharmacology to screen for the potential herbs and active compounds in TCM skin whitening formulas, and explore their possible mechanisms of action. The flow chart of the research performed in this study is shown in Figure 1.

## 2 Methods

### 2.1 Acquisition of Genes Related to Melanin Synthesis

Genes related to melanin synthesis were obtained from the GeneCards database (www.genecards.org) and literature review using “Pigmentation” and “ *Homo sapiens* ” as the initial filtering criteria.

### 2.2 Screening of Herbs With Potential Whitening Effects

Initial screening: Skin whitening prescriptions were searched in *Essential Prescriptions Worth a Thousand Gold*, *Liu Juanzi's Remedies Bequeathed by Ghosts*, *Orthodox Manual of External Diseases*, *Golden Mirror of Medicine*, and the Expert Consensus on the Treatment of Melasma with Chinese Medicine. Venn diagrams were plotted to preliminarily screen for the herbs that appeared repeatedly in the prescriptions. Secondary screening: The active ingredients and potential target genes of the selected herbs were screened from the TCMSP database. The genes common to the melanogenesis‐related gene set in GeneCards database and the potential target gene set were obtained by Venn analysis. The herbs with more than the median number of overlapping target genes were ranked and analyzed further.

### 2.3 Identification of Active Anti‐Melanogenic Compounds

Preliminary screening: The active compounds of the candidate herbs were screened from TCMSP, with oral bioavailability (OB) ≥ 30% and drug‐likeness (DL) ≥ 0.18 as the criteria. OB is defined as the rate and extent to which a drug is absorbed into the body via the intestinal tract, and DL refers to the similarity of a compound to a currently reported drug. The DL value correlates directly to the probability that the compound will be developed as a drug. The target genes of the candidate active compounds were cross‐referenced to the UniProt database, and the compounds corresponding to the target genes not included in the UniProt database were excluded. Secondary screening: The genes common to the gene sets related to melanin synthesis and the target gene sets of the active compounds were obtained, and these overlapping target genes were used for the subsequent analysis. The Cytoscape software was used to draw the network topology of the candidate herbs, active compounds and target genes. The betweenness centrality (BC), closeness centrality (CC) and degree centrality (DC) of each node were calculated using the software algorithm, and the active components with high BC, CC and DC values were selected.

### 2.4 PPI Network Construction

The PPI network of the candidate active components was constructed on the STRING database (https://cn.string‐db.org/), and further visualized by Cytoscape 3.7.2 software.

### 2.5 KEGG Enrichment Analysis

KEGG enrichment analysis was performed for the target genes of each candidate active compound using the Metascape database (metascape.org), restricted to the species “ *Homo sapiens* ”. The histogram of the TOP signaling pathway was plotted.

### 2.6 Molecular Docking

The 2D structures of the active TCM compounds were obtained from the PubChem database, and converted to 3D structures using ChemBio3D Ultra 14.0. Docking proteins were obtained from the PDB and screened for proteins with active pockets and small molecule ligands with “ *Homo sapiens* ”, “X‐RAY DIFFRACTION” and “REFINEBT RESOLUTION ≤ 2.5” as the filters. The small molecule ligands were dissembled from the proteins, and the water molecules and residues such as phosphate and metal atoms were removed from the proteins using PyMoL1.7.2.1 software. The protein activity pockets were obtained with the AutoDock 1.5.6 tool. Molecular docking simulations were performed using AutoDock Vina 1.1.2, and the results were visualized using PyMoL 1.7.2.1 software.

### 2.7 CCK8 Assay

HaCaT and MNT1 cells were cultured in Dulbecco's modified Eagle's medium (DMEM, Gibco, USA) containing 10% fetal bovine serum (FBS, Gibco, USA) and 1% penicillin‐amphotericin (G‐CLONE, China) at 37°C in an incubator with 5% CO2. The cells were treated with different concentrations of acacetin (Selleck, S5318, purity: 99.86%, USA) and quercetin (MedChemExpress, HY‐18085, purity: ≥ 98.0%, USA) for 24 h, and proliferation was detected using the CCK8 assay according to the kit (Servicebio, China) instructions.

### 2.8 HaCaT and MNT1 Co‐Culture

The HaCaT cells were seeded into 6‐well plates at 10% confluency and cultured for 24 h. The MNT1 cells were seeded onto the HaCaT monolayer at 10% confluency. After 24 h, different concentrations of acacetin and quercetin were added to the medium, and the cells were co‐cultured for 24 h.

### 2.9 Masson‐Fontana Melanin Staining

The suitably treated cells were washed with PBS and fixed with 4% paraformaldehyde. After rinsing with double‐distilled water, the cells were stained with Fontana ammonia silver solution (G‐CLONE, Beijing) at 56°C in a water bath for 30 min away from light. The stained cells washed with double‐distilled water and hypo solution, and the melanin granules were observed under an inverted microscope.

### 2.10 Quantitative Real‐Time PCR (qRT‐PCR)

The cultured cells were washed with PBS (Labgic, China) and harvested. Total RNA was extracted using the RNAfast200‐Total RNA Extreme Extraction Kit (Fastagen, China), and reverse transcribed to cDNA using the Reverse Transcription Kit (Vazyme, China). RT‐PCR was performed using a qRT‐PCR kit (Vazyme, China) on the Roche cycler (Eppendorf, Germany) according to the instructions. The primer sequences are listed in the Supporting Information. Relative gene expression was calculated using the 2^−△△Ct^ formula.

### 2.11 Establishment of 3D Melanin Skin Model

The 3D melanin skin model (BIOCELL, PM1021, China) was cultured in EpiKutis solution (BIOCELL, China) and treated with different concentrations of acacetin and quercetin for 3 days. The tissues were fixed with 4% paraformaldehyde (Labgic, China), embedded in paraffin, sectioned, and stained using hematoxylin–eosin (HE) and Fontana Masson ammonia silver solution as per standard protocols.

### 2.12 Statistical Analysis

Histograms were plotted using GraphPad Prism 8.0, and SPSS 22.0 software was used to analyze the data. Continuous variables were expressed as mean ± standard deviation, and compared using t‐test or a non‐parametric test depending whether or not the data conformed to normal distribution. Categorical variables were compared by the chi‐square test. *p* < 0.05 was considered statistically significant. Each experiment was repeated thrice.

## 3 Results

### 3.1 Screening of Herbs With Potential Skin Whitening Effects

**FIGURE 2:**
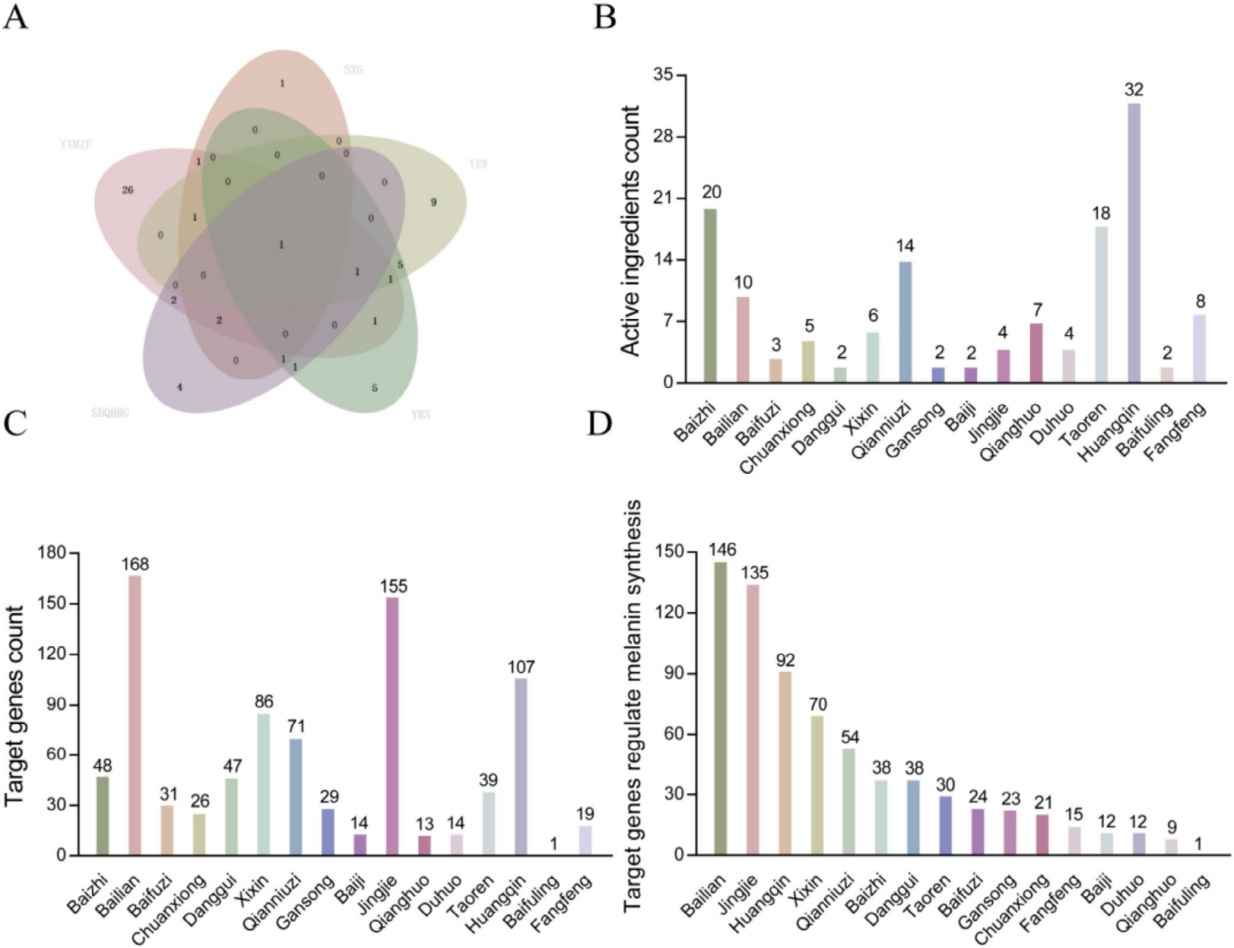
Screening of 16 herbs with potential skin whitening function. (A) The intersecting herbs of five prescriptions identified by the jvenn online platform. (B) The number of active ingredients for each herb. (C) The number of target genes for each herb. (D) Ranking of the number of target genes that may be involved in the regulation of melanin synthesis.

Yuxie Mianzhi Fang, Shexiang Gao, Yurong Wan and Yurong San have been documented as skin whitening prescriptions in *Essential Prescriptions Worth a Thousand Gold*, *Liu Juanzi's Remedies Bequeathed by Ghosts*, *Orthodox Manual of External Diseases* and *Golden Mirror of Medicine*. The Sibai Quban Ruangao is mentioned as a whitening ointment in the expert consensus on the treatment of melasma in Chinese medicine [24]. These five prescriptions contain 36, 7, 18, 16, and 12 herbs respectively (Table S1). *Angelica dahurica* was present in all five prescriptions, and Ampelopsis japonica was present in four prescriptions. *Sauromatum giganteum*, *Conioselinum anthriscoides* ‘Chuanxiong’ (Chuanxiong), *Angelica sinensis* (Oliv.) Diels (Danggui), and *Asarum heterotropoides* F. Schmidt (Xixin) were present in three prescriptions. *Ipomoea nil* (L.) Roth (Qianniuzi), *Nardostachys jatamansi* (D. Don) DC. (Gansong), *Bletilla striata* (Thunb.) Rchb.f. (Baiji), *Nepeta tenuifolia* Benth. (Jingjie), *Hansenia weberbaueriana* (Fedde ex H. Wolff) Pimenov & Kljuykov (Qianghuo), *Angelica pubescens* Maxim. (Duhuo), *Prunus persica* (L.) Batsch (Taoren), *Scutellaria baicalensis* Georgi (Huangqin), *Poria cocos* (Schw.) Wolf (Baifuling), *Saposhnikovia divaricata* (Turcz. ex Ledeb.) Schischk. (Fangfeng), and *Asarum forbesii* Maxim. (Duheng) were present in two prescriptions (Figure 2A and Table 1). All of the above herbs, except *Asarum forbesii* Maxim., were searched in the TCMSP database. The active compounds of the herbs were screened using OB ≥ 30% and DL ≥ 0.18 as the criteria (Table S2), and the target genes corresponding to these active components were also searched. Some of the target genes were not included or validated in the UniProt database, and the corresponding active compounds were excluded from the analysis. Accordingly, there were 20, 10, 3, 5, 2, 6, 14, 2, 2, 4, 7, 4, 18, 32, 2 and 8 active compounds, and 48, 168, 31, 26, 47, 86, 71, 29, 14, 155, 13, 14, 39, 107, 1 and 19 target genes for *Angelica dahurica*, *Ampelopsis japonica*, *Sauromatum giganteum*, *Conioselinum anthriscoides* ‘Chuanxiong’, *Angelica sinensis*, *Asarum heterotropoides* , *Ipomoea nil* , *Nardostachys jatamansi* (D. Don) DC., Bletillae Rhizoma, *Nepeta tenuifolia* Benth., *Hansenia weberbaueriana*, *Angelica pubescens* Maxim., *Prunus persica* , *Scutellaria baicalensis* Georgi, *Poria cocos* and *Saposhnikovia divaricata* respectively (Figure 2B,C and Table S3).

**TABLE 1:** Common herbs in each whitening prescription.

Prescriptions	Common Chinese herbs contain in each prescription
Yuxie Mianzhi Fang, Shexiang Gao, Yurong Wan, Yurong San, Sibai Quban Ruangao	*Angelica dahurica*
Yuxie Mianzhi Fang, Yurong Wan, Yurong San, Sibai Quban Ruangao	*Ampelopsis japonica*
Shexiang Gao, Yurong San, Sibai Quban Ruangao	*Sauromatum giganteum*
Yuxie Mianzhi Fang, Shexiang Gao, Sibai Quban Ruangao	*Conioselinum anthriscoides* ‘Chuanxiong’, *Angelica sinensis*
Yuxie Mianzhi Fang, Shexiang Gao, Yurong Wan	*Asarum heterotropoides*
Yurong San, Sibai Quban Ruangao	*Ipomoea nil*
Yurong Wan, Yurong San	*Nardostachys jatamansi*, *Bletilla striata* , *Nepeta tenuifolia*, *Hansenia weberbaueriana*, *Angelica pubescens*
Yuxie Mianzhi Fang, Sibai Quban Ruangao	*Prunus persica* , *Scutellaria baicalensis*
Yuxie Mianzhi Fang, Yurong San	*Poria cocos*
Yuxie Mianzhi Fang, Yurong Wan	*Saposhnikovia divaricata*
Yuxie Mianzhi Fang, Shexiang Gao	*Asarum forbesii*

The gene regulating melanin production were obtained from GeneCards database. Venn analysis of the melanogenesis‐related genes with the target genes of each active compound showed considerable variation in the number of genes related to melanin production that are potentially regulated by the different herbs. As shown in Figure 2D, *Ampelopsis japonica*, *Nepeta tenuifolia* Benth. and *Scutellaria baicalensis* Georgi regulated a higher number of genes compared to the rest. The herbs with a median or higher number of regulatory genes, including *Ampelopsis japonica*, *Nepeta tenuifolia* Benth., *Scutellaria baicalensis* Georgi, *Asarum heterotropoides* , *Ipomoea nil* , *Angelica dahurica*, *Angelica sinensis* and *Prunus persica* , were selected for further analysis.

### 3.2 Identification of Potential Whitening Active Ingredients

**FIGURE 3:**
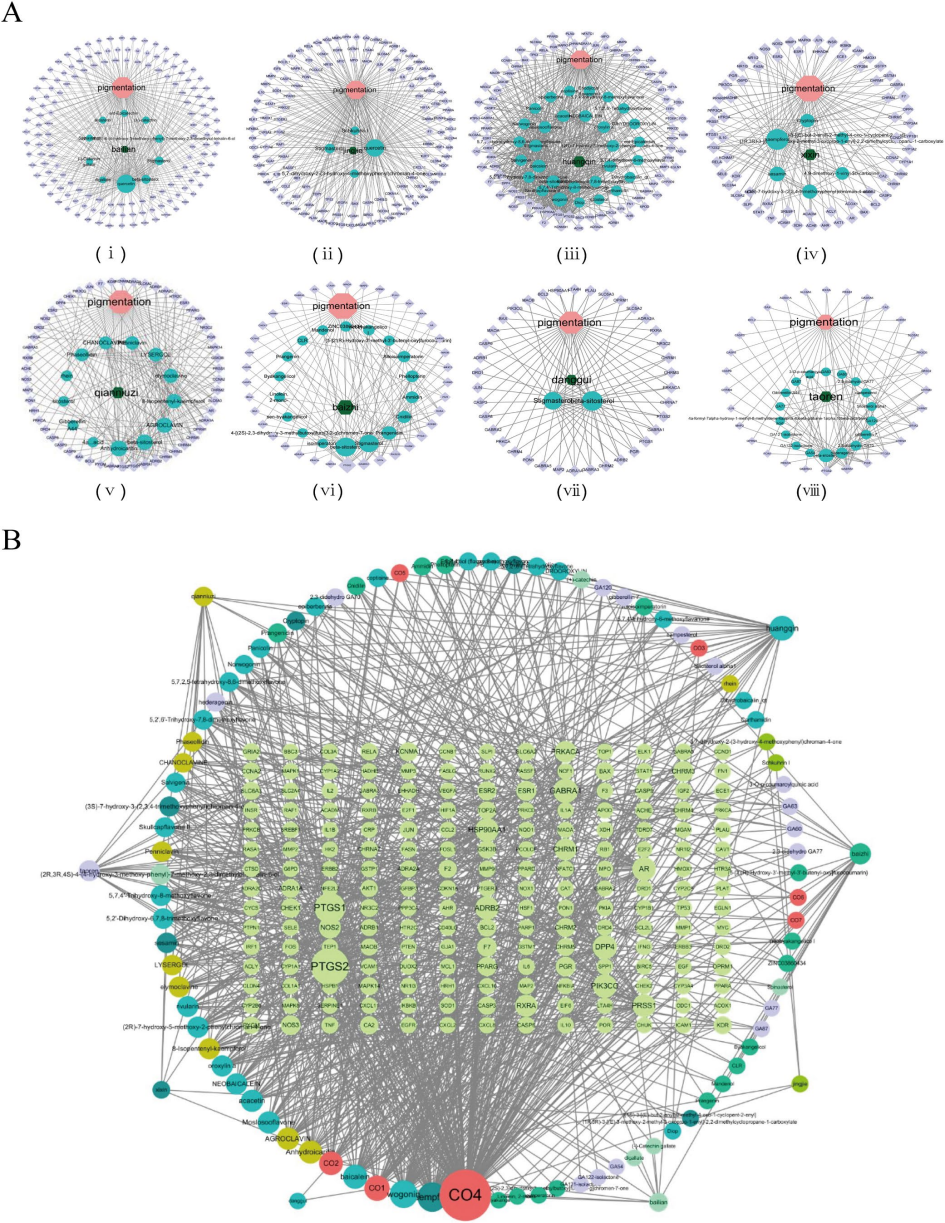
Interaction network of key herbs compounds, diseases and targets. (A) i, ii, iii, iv, v, vi, vii and vii are the complex networks between Bailian, Jingjie, Huangqin, Xixin, Danggui, Baizhi, Qianniuzi, Taoren and pigmentation disease respectively. Red nodes represent disease, dark blue nodes represent herbs, sky blue represent the active ingredients, and purple nodes represent melanin‐related target genes. The edges connected to the node represent their associations. (B) A composite network summarizing the eight herbs (peripheral nodes), active ingredients (circularly arranged nodes) and melanin target genes (rectangularly arranged nodes). CO1: Beta‐sitosterol, CO2: Stigmasterol, CO3: Sitosterol, CO4: Quercetin, CO5: Ent‐Epicatechin, CO6: GibberellinA44, CO7: 4a‐formyl‐7alpha‐hydroxy‐1‐methyl‐8‐methylidene‐4aalpha, 4bbeta‐gibbane‐1alpha, 10beta‐dicarboxylic acid.

The regulatory networks of each candidate herb and the respective active compounds and melanogenesis‐regulated related target genes were constructed using Cytoscape. As shown in Figure 3A, each herbal medicine can regulate multiple target genes through multiple active compounds. Furthermore, the nodes corresponding to the active compounds in each herb varied in size, suggesting different degrees of importance. A composite network of these eight herbs, and their active components and target genes was also constructed (Figure 3B), which showed that different herbs have the same active compounds and may therefore exert their regulatory effects through common pathways. In addition, the different active compounds were associated with the same target genes, suggesting that these herbs may exert pharmacological effects through multiple targets and multiple pathways. To further explore the active components involved in skin whitening, we calculated the BC, CC and DC values of each node. Accordingly, the top 10 active compounds were quercetin, kaempferol, wogonin, beta‐sitosterol, baicalein, stigmasterol, anhydroicaritin, agroclavin, moslosooflavone, and acacetin (Table 2), and their corresponding melanogenesis regulatory target genes are listed in Table S4.

**TABLE 2:** Top 10 active compounds ranked by BC, CC and DC.

Mol‐name	Betweenness centrality	Closeness centrality	Degree centrality
Anhydroicariti	0.02	0.39	0.09
AGROCLAVIN	0.03	0.39	0.08
Moslosooflavone	0.01	0.38	0.07
Acacetin	0.02	0.38	0.07
Quercetin	0.49	0.53	0.16
Kaempferol	0.09	0.42	0.16
Wogonin	0.07	0.41	0.14
Beta‐sitosterol	0.06	0.41	0.12
Baicalein	0.06	0.39	0.10
Stigmasterol	0.05	0.40	0.09

### 3.3 Possible Mechanisms of the Candidate Active Compounds

**FIGURE 4:**
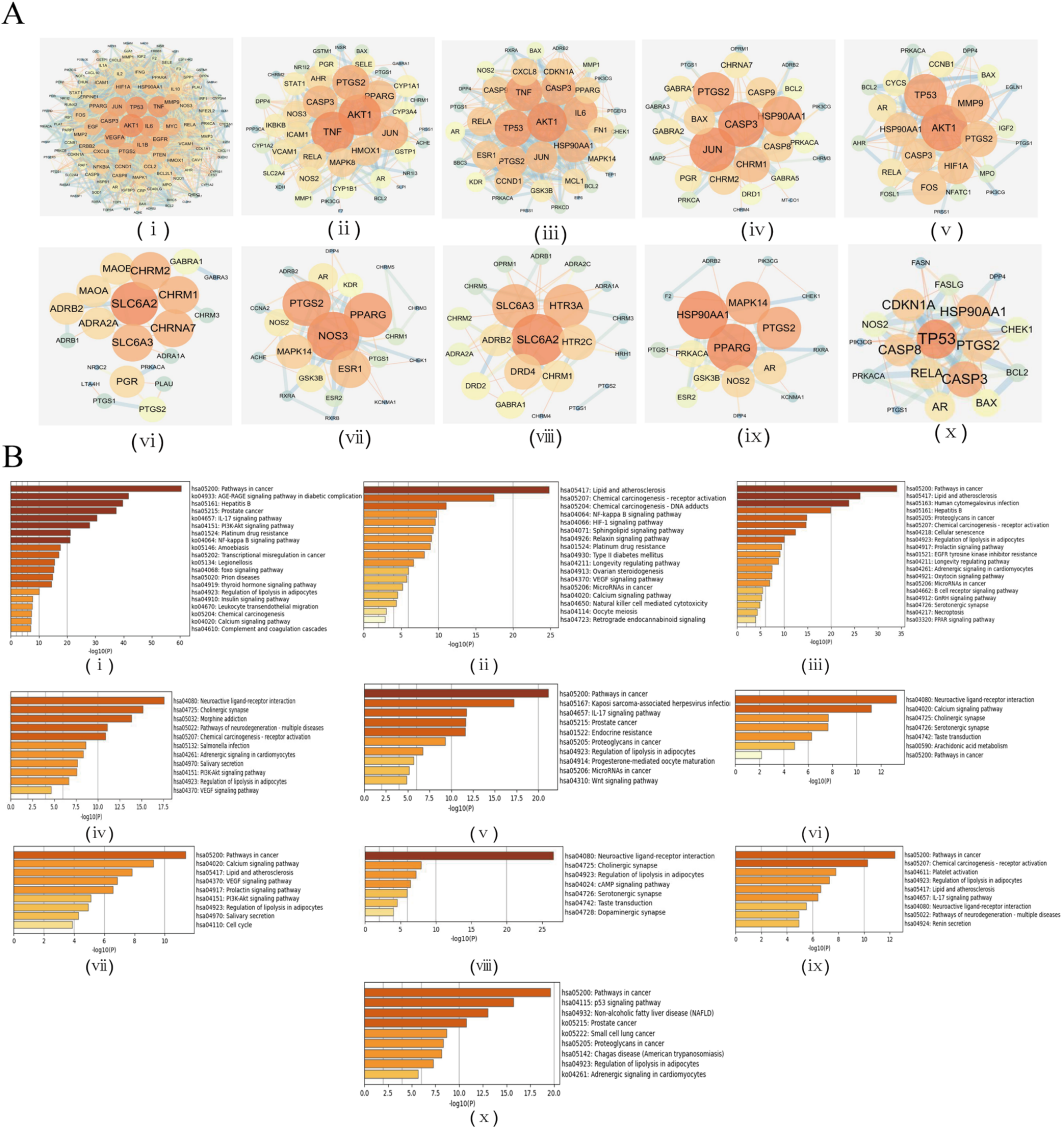
PPI network and pathways of 10 anti‐melanogenic compounds. (A) PPI network construction of target genes involved in the regulation of melanin synthesis for each active component. (B) KEGG enrichment analysis of target genes involved in the regulation of melanin synthesis for each compound. (i) Quercetin, (ii) Kaempferol, (iii) Wogonin, (iv) beta‐sitosterol, (v) Baicalein, (vi) Stigmasterol, (vii) Anhydroicaritin, (viii) AGROCLAVIN, (ix) Moslosooflavone, (x) Acacetin.

The target genes of each active compound were analyzed by PPI network using Cytoscape. As shown in Figure 4A, quercetin had the largest number (124) of target genes, and acacetin had the least number (20) of target genes. The core genes that are potentially regulated by quercetin include JUN, AKT1, IL6, CASP3, IL1B, VEGFA, MMP9, TNF, PTGS2 and EGFR, while the core genes identified for acacetin include TP53, CDKN1A, HSP90AA1, PTGS2, CASP3, RELA and CASP8. To explore the putative signaling pathways regulated by each active component, we performed KEGG enrichment analysis on their target genes through the Metascape database. The top signaling pathways are shown in Figure 4B. Quercetin likely regulates the NF‐κB, AGE‐RAGE, IL‐17 and PI3K‐AKT signaling pathways, while acacetin may be involved in the regulation of P53 and other signaling pathways.

### 3.4 Validation of the Candidate Anti‐Melanogenic Active Compounds

**FIGURE 5:**
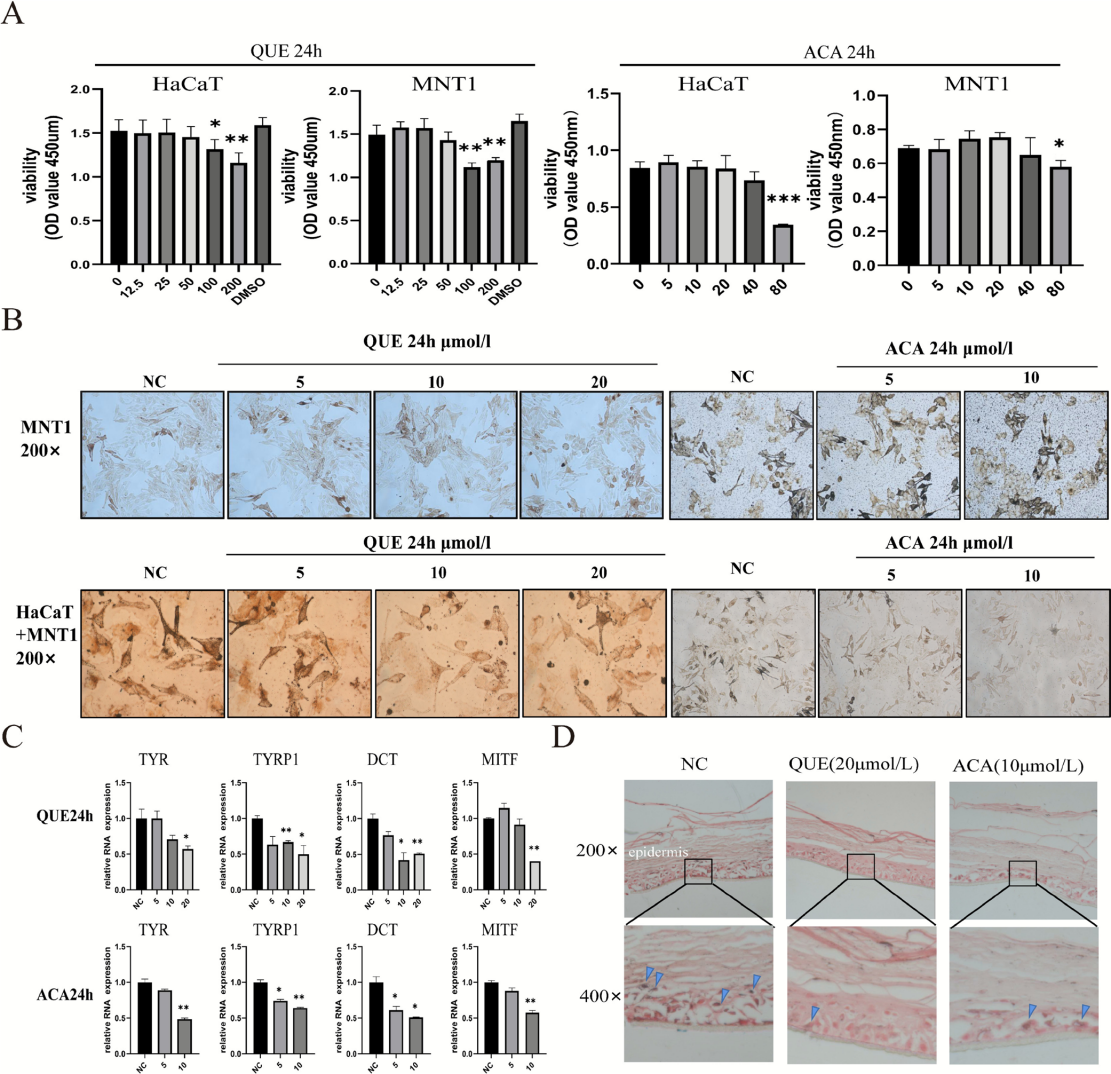
Validation of the effect of candidate active compounds on melanogenesis. (A) The proliferation rates of HaCaT and MNT1 cells treated with different concentrations of quercetin and acacetin for 24 h. (B) Representative images of MNT1 cells and the HaCaT‐MNT1 co‐culture system stained with Fontana‐Masson dye showing the melanin content after treatment with quercetin and acacetin for 24 h (magnification: ×200). (C) TYR, TYRP1, DCT, and MITF mRNA expression in the HaCaT‐MNT1 co‐culture system treated with quercetin and acacetin for 24 h respectively (*p < 0.05, **p < 0.01). (D) Representative images of 3D skin model sections stained with Fontana‐mason dye showing melanin content following treatment with different concentrations of quercetin and acacetin for 72 h (magnification: ×200, upper panels; ×400, lower panels).

We selected quercetin and acacetin for subsequent validation. The effects of both compounds were tested on the human melanoma cell line MNT1 and immortalized human keratinocytes HaCaT. As shown in Figure 5A, quercetin and acacetin had no significant effect on the proliferation rates of the cell lines at doses lower than 50 μmol/L and 40 μmol/L, respectively, after 24 h. To evaluate the effects of quercetin and acacetin on melanin synthesis, the MNT1 cells were treated with different concentrations of each drug for 24 h, and the melanin granules were stained using the Masson‐Fontana method. As shown in Figure 5B, neither compound had any significant effect on melanin production in MNT1 cells. Since keratinocytes regulate melanogenesis through paracrine pathways, we next analyzed whether quercetin and acacetin affect melanin synthesis by targeting these pathways in the keratinocytes. We co‐cultured HaCaT and MNT1 cells to mimic epidermal melanogenic units and treated the co‐culture system with different concentrations of quercetin and acacetin for 24 h. As shown in Figure 5B, high concentrations of quercetin and acacetin inhibited melanin synthesis in the co‐culture system. Furthermore, both compounds downregulated the melanogenesis‐related genes MITF, TYR, TYRP1, and DCT (Figure 5C). To further confirm the skin whitening effects of quercetin and acacetin, we treated 3D melanin skin models with quercetin (20 μmol/L) and acacetin (10 μmol/L) for 72 h, and found that both drugs reduced the melanin amount in 3D skin (Figure 5D). In addition, dose‐dependent inhibition of melanin accumulation by quercetin (5, 10, 20, 40 μmol/L) and acacetin (5, 10, 20, 40 μmol/L) was observed in human foreskin tissue after 3 days of treatment, as assessed by Masson‐Fontana staining (Figure S1).

### 3.5 Quercetin and Acacetin Inhibited the Expression of Paracrine Factors in Keratinocytes

In the PPI network of quercetin and acacetin target genes, PTGS2 was identified as the common hub regulatory gene (Figure 4A). PTGS2 regulates melanogenesis by influencing the paracrine effects of keratinocytes [7], and was significantly downregulated in the HaCaT cells treated with quercetin or acacetin. In addition, both quercetin and acacetin also inhibited the expression of ET‐1 and VEGF, which are important paracrine factors regulating melanogenesis. The NF‐κB pathway lies upstream of PTGS2, VEGF and ET‐1. To determine whether quercetin and acacetin regulate this pathway, we tested their affinity for TLR4, CHUK and RELA, the upstream and downstream key genes in the NF‐κB signaling pathway, through molecular docking simulations. Based on intermolecular binding energy (≤ −6 kcal/mol generally indicates good affinity), quercetin and acacetin showed good affinity to TLR4, CHUK and RELA. As shown in Figure S2, the binding energies of quercetin to TLR4, CHUK and RELA were −9 kcal/mol, −8.9 kcal/mol and −8.2 kcal/mol respectively, and that of acacetin to TLR4, CHUK and RELA were −9.4 kcal/mol, −9.2 kcal/mol and −8.6 kcal/mol respectively. These results suggest that quercetin and acacetin may inhibit the paracrine effects of keratinocytes by targeting the NF‐κB pathway. Consistently, further docking with NF‐κB downstream effectors (PTGS2, VEGF, ET‐1, IL‐1, TNF) demonstrated favorable binding affinities (≤ −6.0 kcal/mol), reinforcing their inhibitory effects on keratinocyte‐derived paracrine signaling (Figure S3).

Moreover, to delineate herb‐specific contributions beyond the ubiquitous quercetin, we extended docking analyses to one representative core compound from each of the eight prioritized herbs, selected by a composite scoring strategy integrating network centralities (closeness, betweenness, and degree centralities) and specificity (defined as the frequency of occurrence across herbs). These herb‐specific compounds also exhibited favorable binding affinities with NF‐κB pathway targets (Figure S4), supporting the notion that anti‐inflammatory Chinese herbal medicines may exert potential whitening effects by regulating keratinocyte‐derived paracrine signaling through the NF‐κB pathway.

## 4 Discussion

Based on network pharmacology, we identified 8 potential Chinese herbal medicines (*Ampelopsis japonica*, *Nepeta tenuifolia* Benth., *Scutellaria baicalensis* Georgi, *Asarum heterotropoides* , *Ipomoea nil* , *Angelica dahurica*, *Angelica sinensis* and *Prunus persica* ) and 10 potential active compounds (quercetin, kaempferol, wogonin, beta‐sitosterol, baicalein, stigmasterol, anhydroicaritin, agroclavin, moslosooflavone, acacetin) from five prescriptions. In vitro experiments further confirmed that quercetin and acacetin can inhibit skin pigmentation.

Among the eight candidate herbs screened in this study, some have been shown to inhibit melanin production in vitro or in vivo. For example, aqueous and ethanolic extracts of *Angelica dahurica* and *Ampelopsis japonica* can inhibit tyrosinase activity in vitro [29]. In addition, *Scutellaria baicalensis* Georgi extract inhibited tyrosinase activity and melanin synthesis in B16F10 cells [30, 31], and the extract of *Angelica sinensis* decreased melanogenesis in co‐culture system of human melanoma and keratinocytes cells [32]. Likewise, there is evidence regarding the anti‐melanogenic effects of the 10 candidate active compounds. For example, quercetin and baicalein exhibited an inhibitory effect on melanogenesis in mouse cells [33, 34], and kaempferol inhibits melanin synthesis by acting on tyrosinase and MITF [35]. Wogonin been shown to exert anti‐melanogenic activity in B16F10 cells as well as human primary melanocytes by inhibiting tyrosinase synthesis and melanin transport [36]. In addition, beta‐sitosterol present in the extracts of *Annona reticulata* L. inhibits melanin synthesis through the P38 pathway, and acacetin can inhibit tyrosinase activity in vitro [37]. These reports indirectly confirm that network pharmacology is a reliable method for exploring skin whitening drugs or active compounds. Other unreported Chinese herbal medicines and their active components may be potentially anti‐melanogenic. We preliminarily explored the possible mechanisms of the candidate herbs and compounds through PPI and KEGG analysis, which will have to be experimentally validated.

The anti‐melanogenic effects of quercetin and acacetin were demonstrated using cellular and tissue models. The role of quercetin in melanin synthesis is currently controversial; while some studies have shown that quercetin inhibits melanogenesis [38, 39], others have reported a pro‐melanogenic effect [40, 41]. Most studies have only explored the direct effect of quercetin on melanocytes and ignored the influence of keratinocytes in the epidermal melanocyte unit. In our study, quercetin inhibited pigmentation in a 3D skin model but did not have a direct effect on melanogenesis in the MNT1 cells. However, quercetin inhibited melanogenesis in the MNT1‐HaCaT co‐culture system, suggesting that it blocks the melanogenic pathway indirectly via keratinocytes. ET‐1, VEGF and PTGS2 are among the paracrine factors secreted by keratinocytes to regulate melanogenesis [7], and were downregulated in the HaCaT cells following quercetin treatment. The NF‐κB signaling pathway is involved in the regulation of melanogenesis [42]. Ramie extract can inhibit inflammation‐induced skin hyperpigmentation in mice through the NF‐κB pathway [43]. Furthermore, sulforaphane can reduce melanin synthesis in melanocytes by inhibiting ET‐1 and PGE2 synthesis in the co‐cultured HaCaT cells by targeting the NF‐κB pathway [44]. Several studies have shown that the NF‐κB pathway can upregulate PTGS2, ET‐1 and VEGF [45, 46, 47]. Molecular docking experiments showed good affinity of quercetin with multiple regulatory molecules in the NF‐κB pathway, indicating that quercetin most likely downregulates PTGS2, ET‐1 and VEGF by inhibiting NF‐κB signaling, which in turn represses the paracrine effects of keratinocytes. Nevertheless, keratin‐forming cells express other paracrine factors that regulate melanogenesis, and it remains to be ascertained whether quercetin can affect melanogenesis through other pathways or molecules.

Some studies have shown that acacetin can directly act on melanocytes to inhibit melanogenesis [48, 49]. However, we found that acacetin inhibited melanogenesis indirectly by blocking the paracrine effects of keratinocytes. Acacetin has been reported to have a significant inhibitory effect on the NF‐κB pathway [50]. Consistent with this, molecular docking revealed good affinity of acacetin with several regulatory molecules in this pathway. Therefore, acacetin may also suppress the paracrine effects of keratinocytes by downregulating PTGS2, ET‐1 and VEGF via the NF‐κB signaling pathway. A limitation of this study is the lack of skin permeability and bioavailability assessment of the identified compounds, parameters essential for topical application. Future studies should address this by employing in vitro penetration assays or in vivo pharmacokinetic evaluation to confirm their translational potential.

## 5 Conclusions

At present, most studies on the whitening activity of flavonoids like quercetin and acacetin have focused on the inhibition of tyrosinase and melanin synthesis via MITF. However, it is unclear whether the anti‐inflammatory effects of these flavonoids exerted via the NF‐κB pathway can inhibit melanogenesis. Using network pharmacology and in vitro experiments, we found that Chinese herbal medicine with strong anti‐inflammatory activity may inhibit melanogenesis by regulating the paracrine effects of keratinocytes through the NF‐κB signaling pathway. Therefore, our findings provide preclinical evidence that the NF‐κB pathway is a promising target for reversing skin hyperpigmentation.

## Author Contributions

Conceptualization, Methodology, Funding acquisition: Ling Jiang. Data analysis, Visualization, Validation, Manuscript writing: Xinxin Lei, Yixuan Liang. Review and Editing: Chen Jing, Chuhan Fu, Xixia Dai.

## Ethics Statement

The design and methods of the research were approved by the Ethics Committee of the Third Xiangya Hospital of Central South University (approval documents: No. 2021‐S137).

## Consent

The authors have nothing to report.

## Conflicts of Interest

The authors declare no conflicts of interest.

## Supporting information


**Figure S1:** Masson‐Fontana staining of human foreskin tissue treated with quercetin (QUE, 5–40 μmol/L) or acacetin (ACA, 5–40 μmol/L) for 3 days, showing melanin content and corresponding quantitative analysis.
**Figure S2:** Molecular docking models of quercetin and acacetin with their predicted protein targets.
**Figure S3:** Molecular docking models of quercetin and acacetin with NF‐κB downstream effectors, including PTGS2, VEGF (VEGFR), ET‐1 (EDNR), IL‐1 (IL1R), and TNF (TNFR).
**Figure S4:** Molecular docking models of herb‐specific core compounds from eight prioritized herbs with NF‐κB pathway targets (TLR4, CHUK, RELA) and PTGS2.

**Table S1:** The composition of each skin whitening prescription.
**Table S2:** Active ingredients of 16 Chinese herbs. (Initial screening: OB ≥ 30%, DL ≥ 0.18).
**Table S3:** Target genes for Chinese herbs.
**Table S4:** Melanogenesis‐related target genes for 10 candidate active ingredients.

## Data Availability

All data generated or analyzed during this study are included in this published article.
