# Supplementary material for: Network Pharmacology Approach to Explore the Skin‐Lightening Compounds and Potential Mechanisms of Chinese Herbal Medicines
Source: J Cosmet Dermatol. 2025 Dec 5;24(12):e70562. doi: 10.1111/jocd.70562 (PMC12680926; doi:10.1111/jocd.70562)
Supplement: Supplementary file 1 — Figure S1: Masson‐Fontana staining of human foreskin tissue treated with quercetin (QUE, 5–40 μmol/L) or acacetin (ACA, 5–40 μmol/L) for 3 days, showing melanin content and corresponding quantitative analysis. Figure S2: Molecular docking models of quercetin and acacetin with their predicted protein targets. Figure S3: Molecular docking models of quercetin and acacetin with NF‐κB downstream effectors, including PTGS2, VEGF (VEGFR), ET‐1 (EDNR), IL‐1 (IL1R), and TNF (TNFR). Figure S4: Molecular docking models of herb‐specific core compounds from eight prioritized herbs with NF‐κB pathway targets (TLR4, CHUK, RELA) and PTGS2. [file JOCD-24-e70562-s002.doc]

**Figure S1.** Masson-Fontana staining of human foreskin tissue treated with quercetin (QUE, 5–40 μmol/L) or acacetin (ACA, 5–40 μmol/L) for 3 days, showing melanin content and corresponding quantitative analysis.


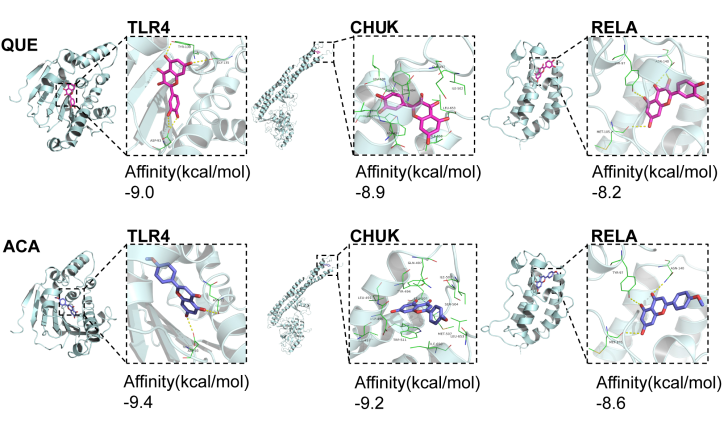


**Figure S2.** Molecular docking models of quercetin and acacetin with their predicted protein targets.

**
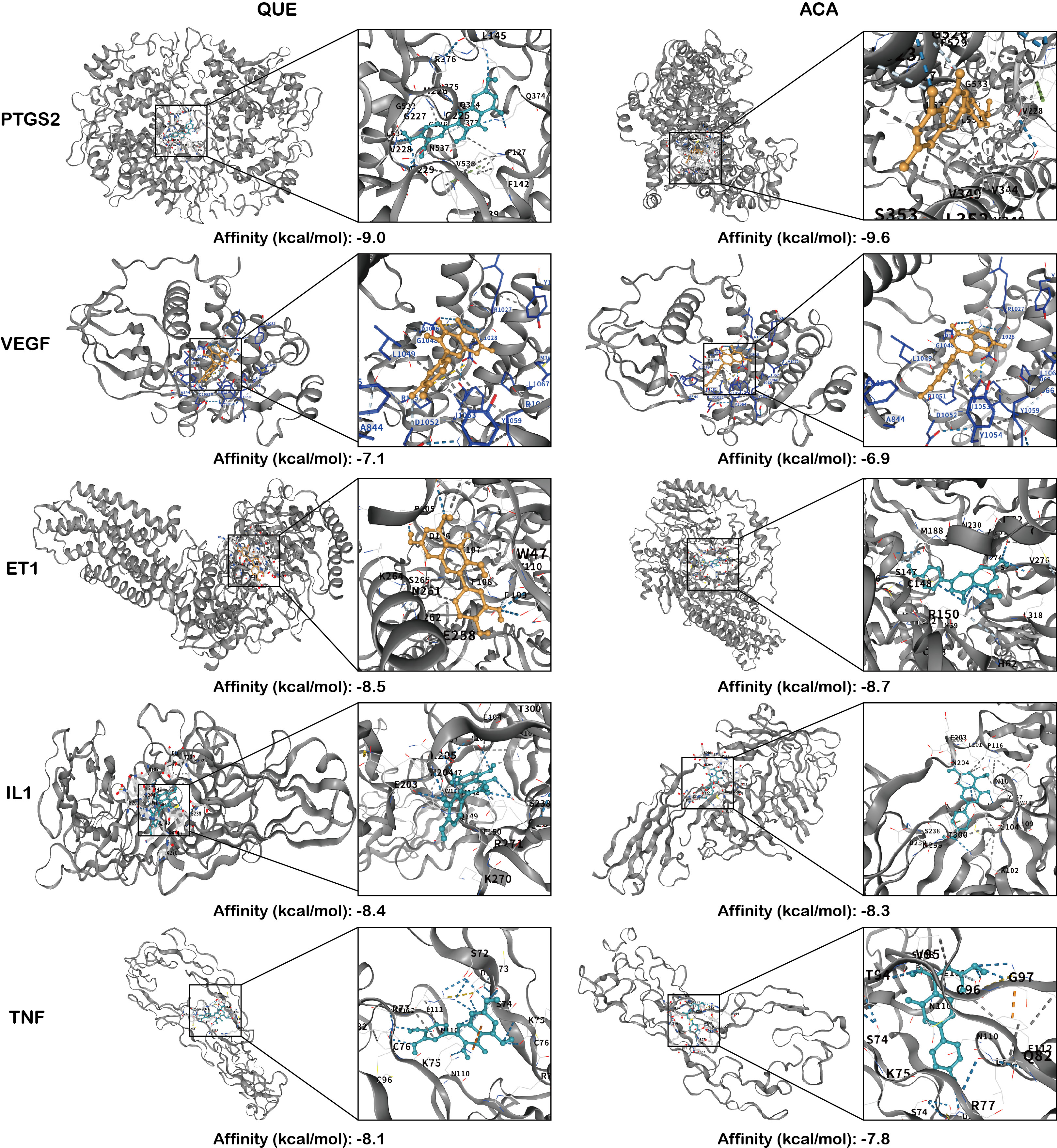
**

**Figure S3.** Molecular docking models of quercetin and acacetin with NF-κB downstream effectors, including PTGS2, VEGF (VEGFR), ET-1 (EDNR), IL-1 (IL1R), and TNF (TNFR).


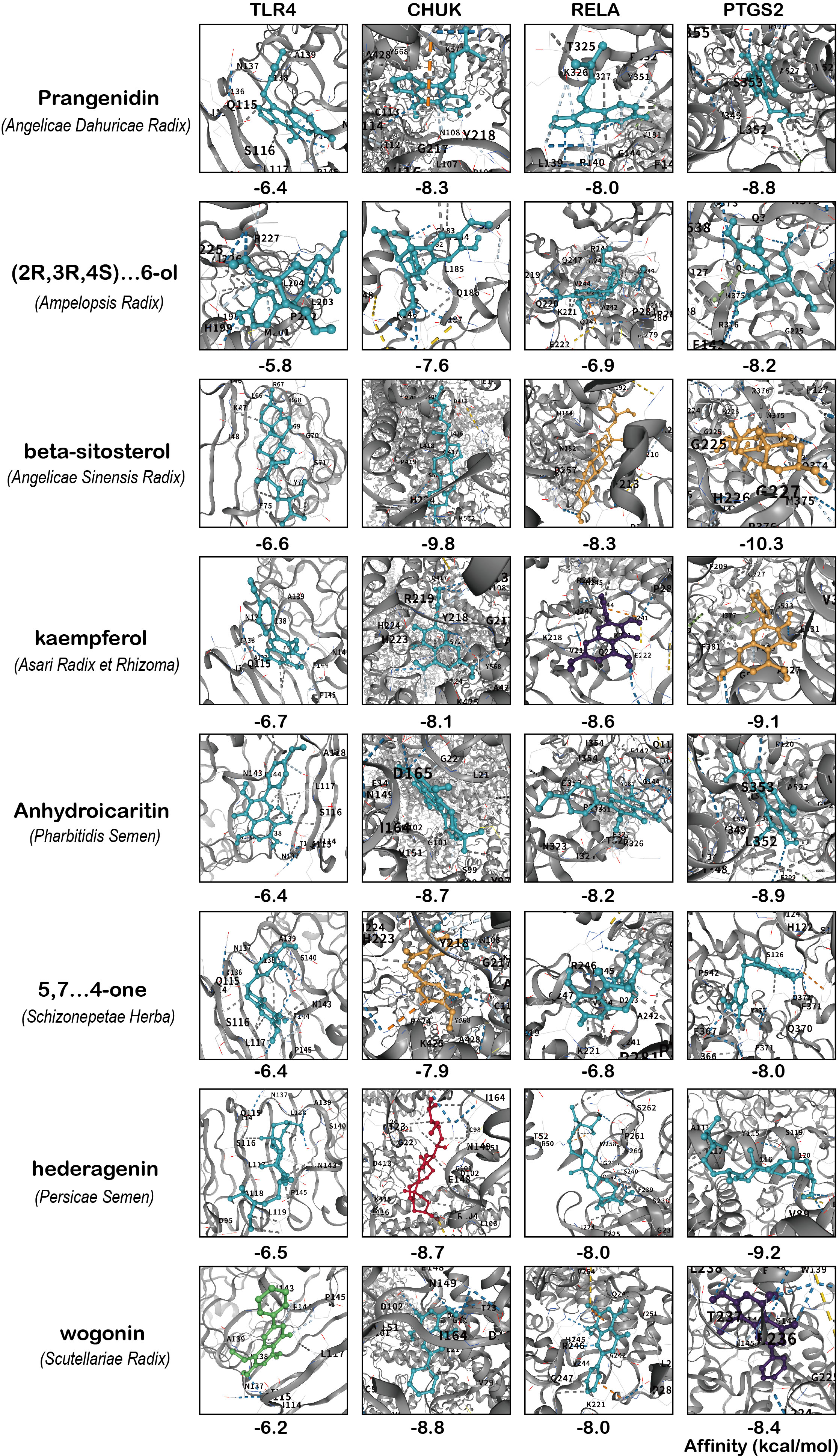


**Figure S4.** Molecular docking models of herb-specific core compounds from eight prioritized herbs with NF-κB pathway targets (TLR4, CHUK, RELA) and PTGS2. Abbreviations: 2R,3R,4S…6-ol, (2R,3R,4S)-4-(4-hydroxy-3-methoxy-phenyl)-7-methoxy-2,3-dimethylol-tetralin-6-ol; 5,7…4-one, 5,7-dihydroxy-2-(3-hydroxy-4-methoxyphenyl)chroman-4-one.
