# Supplementary material for: Network Pharmacology Approach to Explore the Skin‐Lightening Compounds and Potential Mechanisms of Chinese Herbal Medicines
Source: J Cosmet Dermatol. 2025 Dec 5;24(12):e70562. doi: 10.1111/jocd.70562 (PMC12680926; doi:10.1111/jocd.70562)
Supplement: Supplementary file 2 — Table S1: The composition of each skin whitening prescription. Table S2: Active ingredients of 16 Chinese herbs. (Initial screening: OB ≥ 30%, DL ≥ 0.18). Table S3: Target genes for Chinese herbs. Table S4: Melanogenesis‐related target genes for 10 candidate active ingredients. [file JOCD-24-e70562-s001.doc]

**Table S1** The composition of each skin whitening prescription.

| **Prescriptions** | **Main components: TCM** |
| --- | --- |
| Yuxie Mianzhi Fang (36 kinds of herbs) | Conioselinum anthriscoides 'Chuanxiong', Angelica sinensis, Trichosanthes cucumeroides, Cuscuta chinensis, Magnolia denudata, Asarum heterotropoides, Polygonatum odoratum, Phytolacca acinosa, Angelica dahurica, Saposhnikovia divaricata, Prunus persica, Magnolia officinalis, Agastache rugosa, Kitagawia praeruptora, Neolitsea cassia, Benincasa hispida, Pinellia ternata, Ampelopsis japonica, Aristolochia debilis, Prunus armeniaca, Ligusticum chuanxiong Hort. (Miwu), Inula japonica, Asarum forbesii, Musk, Poria cocos, Zanthoxylum bungeanum (Qinjiao), Pulsatilla chinensis, Fraxinus chinensis, Pollia japonica, Zanthoxylum bungeanum (Shujiao), Brassica rapa seed, Actaea cimicifuga, Scutellaria baicalensis Georgi, Vincetoxicum atratum, Gardenia flower, Trichosanthes kirilowii seed |
| Shexiang Gao (7 kinds of herbs) | Angelica sinensis, Sauromatum giganteum, Conioselinum anthriscoides 'Chuanxiong', Angelica dahurica, Paeonia lactiflora, Asarum heterotropoides, Asarum forbesii |
| Yurong Wan (18 kinds of herbs) | Nardostachys jatamansi, Kaempferia galanga, Asarum heterotropoides, Angelica dahurica, Ampelopsis japonica, Bletilla striata, Saposhnikovia divaricata, Nepeta tenuifolia Benth., Bombyx Batryticatus, Gardenia jasminoides, Conioselinum anthriscoides, Gastrodia elata, Hansenia weberbaueriana, Angelica pubescens, Chrysanthemum × morifolium, Zanthoxylum bungeanum, Ziziphus jujuba, Santalum album |
| Yurong San (16 kinds of herbs) | Ipomoea nil, Ampelopsis japonica, Actaea cimicifuga, Nardostachys jatamansi, Bletilla striata, Nymphaea alba, Angelica dahurica, Atractylodes macrocephala, Poria cocos, Nepeta tenuifolia Benth., Angelica pubescens Maxim., Hansenia weberbaueriana, Sauromatum giganteum, Lablab purpureus subsp. purpureus, Saposhnikovia divaricata, Syringa oblata var.affinis |
| Sibai Quban Ruangao (12 kinds of herbs) | Calamus draco, Panax notoginseng, Semen armeniacae, Prunus persica, Ipomoea nil, Angelica dahurica, Sauromatum giganteum, Angelica sinensis, Coix lacryma-jobi, Ampelopsis japonica, [Scutellaria baicalensis](https://mpns.science.kew.org/mpns-portal/plantDetail?plantId=188938&query=黄芩&filter=&fuzzy=false&nameType=all&dbs=wcs), Conioselinum anthriscoides 'Chuanxiong' |

**Table S2** Active ingredients of 16 Chinese herbs. (Initial screening: OB≥30%, DL≥0.18)

| Herb name | Mol ID | Molecule Name | OB (%) | DL |
| --- | --- | --- | --- | --- |
| Angelicae Dahuricae Radix (Baizhi) | MOL001494 | mandenol | 42.00 | 0.19 |
|  | MOL001939 | alloisoimperatorin | 34.80 | 0.22 |
|  | MOL001941 | ammidin | 34.55 | 0.22 |
|  | MOL001942 | isoimperatorin | 45.46 | 0.23 |
|  | MOL001956 | cnidilin | 32.69 | 0.28 |
|  | MOL002883 | ethyl oleate (NF) | 32.40 | 0.19 |
|  | MOL005789 | neobyakangelicol | 36.18 | 0.31 |
|  | MOL005792 | 5-…furocoumarin | 42.85 | 0.26 |
|  | MOL005800 | byakangelicol | 41.42 | 0.36 |
|  | MOL005802 | propyleneglycol monoleate | 37.60 | 0.26 |
|  | MOL005806 | 4-…7-one | 39.99 | 0.29 |
|  | MOL005807 | sen-byakangelicol | 58.00 | 0.61 |
|  | MOL000358 | beta-sitosterol | 36.91 | 0.75 |
|  | MOL000449 | stigmasterol | 43.83 | 0.76 |
|  | MOL000953 | CLR | 37.87 | 0.68 |
|  | MOL001506 | supraene | 33.55 | 0.42 |
|  | MOL001749 | ZINC03860434 | 43.59 | 0.35 |
|  | MOL002644 | phellopterin | 40.19 | 0.28 |
|  | MOL003588 | prangenidin | 36.31 | 0.22 |
|  | MOL003791 | linolein, 2-mono- | 37.28 | 0.30 |
|  | MOL007514 | methyl icosa-11,14-dienoate | 39.67 | 0.23 |
|  | MOL013430 | prangenin | 43.60 | 0.29 |
| Ampelopsis Radix (Bailian) | MOL003283 | (2R,3R,4S)…6-ol | 66.51 | 0.39 |
|  | MOL000358 | beta-sitosterol | 36.91 | 0.75 |
|  | MOL000359 | sitosterol | 36.91 | 0.75 |
|  | MOL004355 | spinasterol | 42.98 | 0.76 |
|  | MOL000449 | stigmasterol | 43.83 | 0.76 |
|  | MOL000492 | (+)-catechin | 54.83 | 0.24 |
|  | MOL000569 | digallate | 61.85 | 0.26 |
|  | MOL006504 | (-)-catechin gallate | 53.57 | 0.75 |
|  | MOL000073 | ent-epicatechin | 48.96 | 0.24 |
|  | MOL000098 | quercetin | 46.43 | 0.28 |
| Typhonii Rhizoma (Baifuzi) | MOL001494 | mandenol | 42.00 | 0.19 |
|  | MOL000358 | beta-sitosterol | 36.91 | 0.75 |
|  | MOL000359 | sitosterol | 36.91 | 0.75 |
| Chuanxiong Rhizoma (Chuanxiong) | MOL001494 | mandenol | 42.00 | 0.19 |
|  | MOL002135 | myricanone | 40.60 | 0.51 |
|  | MOL002140 | perlolyrine | 65.95 | 0.27 |
|  | MOL002151 | senkyunone | 47.66 | 0.24 |
|  | MOL002157 | wallichilide | 42.31 | 0.71 |
|  | MOL000359 | sitosterol | 36.91 | 0.75 |
|  | MOL000433 | FA | 68.96 | 0.71 |
| Angelicae Sinensis Radix (Danggui) | MOL000358 | beta-sitosterol | 36.91 | 0.75 |
|  | MOL000449 | stigmasterol | 43.83 | 0.76 |
| Asari Radix et Rhizoma (Xixin) | MOL012140 | 4,9-dimethoxy-1-vinyl-$b-carboline | 65.30 | 0.19 |
|  | MOL001460 | cryptopin | 78.74 | 0.72 |
|  | MOL001558 | sesamin | 56.55 | 0.83 |
|  | MOL002501 | 1S…1-carboxylate | 62.52 | 0.31 |
|  | MOL002962 | (3S) …4-one | 48.23 | 0.33 |
|  | MOL000422 | kaempferol | 41.88 | 0.24 |
| Pharbitidis Semen (Qianniuzi) | MOL001348 | gibberellin 17 | 94.64 | 0.49 |
|  | MOL001349 | 4a-… acid | 88.60 | 0.46 |
|  | MOL001351 | gibberellin A44 | 101.61 | 0.54 |
|  | MOL002268 | rhein | 47.07 | 0.28 |
|  | MOL000358 | beta-sitosterol | 36.91 | 0.75 |
|  | MOL000359 | sitosterol | 36.91 | 0.75 |
|  | MOL004373 | anhydroicaritin | 45.41 | 0.44 |
|  | MOL003542 | 8-Isopentenyl-kaempferol | 38.04 | 0.39 |
|  | MOL000457 | phaseollidin | 52.04 | 0.53 |
|  | MOL005236 | gibberellin | 81.59 | 0.53 |
|  | MOL005240 | nimbolidin B | 30.22 | 0.61 |
|  | MOL005246 | gibberellin A20 | 94.93 | 0.49 |
|  | MOL005247 | gibberellin A26 | 57.75 | 0.58 |
|  | MOL005248 | gibberellin A29 | 92.38 | 0.53 |
|  | MOL005251 | gibberellin glucoside I_qt | 81.86 | 0.53 |
|  | MOL005252 | gibberellin glucosideII | 37.40 | 0.48 |
|  | MOL005252 | gibberellin glucosideII | 37.40 | 0.48 |
|  | MOL005257 | penniclavin | 48.15 | 0.31 |
|  | MOL005260 | chanoclavine | 62.64 | 0.18 |
|  | MOL005261 | lysergol | 48.11 | 0.27 |
|  | MOL005266 | agroclavin | 47.71 | 0.24 |
|  | MOL005267 | elymoclavine | 72.87 | 0.27 |
|  | MOL000554 | gallic acid-3-O-(6'-O-galloyl)-glucoside | 30.25 | 0.67 |
| Nardostachyos Radix et Rhizoma (Gansong) | MOL001040 | (2R) …4-one | 42.36 | 0.21 |
|  | MOL007088 | cryptotanshinone | 52.34 | 0.40 |
| Bletillae Rhizoma (Baiji) | MOL005755 | 1-…2,7-diol | 54.18 | 0.55 |
|  | MOL005770 | bletlol A | 54.43 | 0.55 |
|  | MOL005773 | blespirol | 43.74 | 0.86 |
| Schizonepetae Herba (Jingjie) | MOL011856 | schkuhrin I | 54.45 | 0.52 |
|  | MOL005100 | 5,7…4-one | 47.74 | 0.27 |
|  | MOL000098 | quercetin | 46.43 | 0.28 |
|  | MOL000449 | stigmasterol | 43.83 | 0.76 |
| Notopterygii Rhizoma et Radix (Qianghuo) | MOL011963 | 8-geranoxy-5-methoxypsoralen | 40.97 | 0.50 |
|  | MOL011969 | demethylfuropinnarin | 41.31 | 0.21 |
|  | MOL011971 | diversoside_qt | 67.57 | 0.31 |
|  | MOL011975 | notoptol | 62.97 | 0.48 |
|  | MOL001951 | bergaptin | 41.73 | 0.42 |
|  | MOL004792 | nodakenin | 57.12 | 0.69 |
|  | MOL001942 | isoimperatorin | 45.46 | 0.23 |
|  | MOL002644 | phellopterin | 40.19 | 0.28 |
| Angelicae Pubescentis Radix (Duhuo) | MOL001942 | isoimperatorin | 45.46 | 0.23 |
|  | MOL003608 | O-acetylcolumbianetin | 60.04 | 0.26 |
|  | MOL004778 | (1R,2R)…enoate | 46.03 | 0.34 |
|  | MOL004782 | (1R,2R)…methylbutanoate | 45.19 | 0.34 |
|  | MOL004792 | nodakenin | 57.12 | 0.69 |
| Persicae Semen (Taoren) | MOL001323 | sitosterol alpha1 | 43.28 | 0.78 |
|  | MOL001328 | 2,3-didehydro GA70 | 63.29 | 0.50 |
|  | MOL001329 | 2,3-didehydro GA77 | 88.08 | 0.53 |
|  | MOL001339 | GA119 | 76.36 | 0.49 |
|  | MOL001340 | GA120 | 84.85 | 0.45 |
|  | MOL001342 | GA121-isolactone | 72.70 | 0.54 |
|  | MOL001343 | GA122 | 64.79 | 0.50 |
|  | MOL001344 | GA122-isolactone | 88.11 | 0.54 |
|  | MOL001348 | gibberellin 17 | 94.64 | 0.49 |
|  | MOL001349 | 4a…acid | 88.60 | 0.46 |
|  | MOL001350 | GA30 | 61.72 | 0.54 |
|  | MOL001351 | gibberellin A44 | 101.61 | 0.54 |
|  | MOL001352 | GA54 | 64.21 | 0.53 |
|  | MOL001353 | GA60 | 93.17 | 0.53 |
|  | MOL001355 | GA63 | 65.54 | 0.54 |
|  | MOL001358 | gibberellin 7 | 73.80 | 0.50 |
|  | MOL001360 | GA77 | 87.89 | 0.53 |
|  | MOL001361 | GA87 | 68.85 | 0.57 |
|  | MOL001368 | 3-O-p-coumaroylquinic acid | 37.63 | 0.29 |
|  | MOL001371 | Populoside_qt | 108.89 | 0.20 |
|  | MOL000296 | hederagenin | 36.91 | 0.75 |
|  | MOL000358 | beta-sitosterol | 36.91 | 0.75 |
|  | MOL000493 | campesterol | 37.58 | 0.71 |
| Scutellariae Radix (Huangqin) | MOL001689 | acacetin | 34.97 | 0.24 |
|  | MOL000173 | wogonin | 30.68 | 0.23 |
|  | MOL000228 | (2R)-7-hydroxy-5-methoxy-2-phenylchroman-4-one | 55.23 | 0.20 |
|  | MOL002714 | baicalein | 33.52 | 0.21 |
|  | MOL002908 | 5,8,2'-Trihydroxy-7-methoxyflavone | 37.01 | 0.27 |
|  | MOL002909 | 5,7,2,5-tetrahydroxy-8,6-dimethoxyflavone | 33.82 | 0.45 |
|  | MOL002910 | carthamidin | 41.15 | 0.24 |
|  | MOL002911 | 2,6,2',4'-tetrahydroxy-6'-methoxychaleone | 69.04 | 0.22 |
|  | MOL002913 | dihydrobaicalin_qt | 40.04 | 0.21 |
|  | MOL002914 | eriodyctiol (flavanone) | 41.35 | 0.24 |
|  | MOL002915 | salvigenin | 49.07 | 0.33 |
|  | MOL002917 | 5,2',6'-trihydroxy-7,8-dimethoxyflavone | 45.05 | 0.33 |
|  | MOL002925 | 5,7,2',6'-Tetrahydroxyflavone | 37.01 | 0.24 |
|  | MOL002926 | dihydrooroxylin A | 38.72 | 0.23 |
|  | MOL002927 | skullcapflavone II | 69.51 | 0.44 |
|  | MOL002928 | oroxylin a | 41.37 | 0.23 |
|  | MOL002932 | panicolin | 76.26 | 0.29 |
|  | MOL002933 | 5,7,4'-trihydroxy-8-methoxyflavone | 36.56 | 0.27 |
|  | MOL002934 | neobaicalein | 104.34 | 0.44 |
|  | MOL002937 | dihydrooroxylin | 66.06 | 0.23 |
|  | MOL000358 | beta-sitosterol | 36.91 | 0.75 |
|  | MOL000359 | sitosterol | 36.91 | 0.75 |
|  | MOL000525 | norwogonin | 39.40 | 0.21 |
|  | MOL000073 | ent-epicatechin | 48.96 | 0.24 |
|  | MOL000449 | stigmasterol | 43.83 | 0.76 |
|  | MOL001458 | coptisine | 30.67 | 0.86 |
|  | MOL001490 | bis[(2S)-2-ethylhexyl] benzene-1,2-dicarboxylate | 43.59 | 0.35 |
|  | MOL001506 | supraene | 33.55 | 0.42 |
|  | MOL002879 | diop | 43.59 | 0.39 |
|  | MOL002897 | epiberberine | 43.09 | 0.78 |
|  | MOL008206 | moslosooflavone | 44.09 | 0.25 |
|  | MOL010415 | 11,13-eicosadienoic acid, methyl ester | 39.28 | 0.23 |
|  | MOL012245 | 5,7,4'-trihydroxy-6-methoxyflavanone | 36.63 | 0.27 |
|  | MOL012246 | 5,7,4'-trihydroxy-8-methoxyflavanone | 74.24 | 0.26 |
|  | MOL012266 | rivularin | 37.94 | 0.37 |
| Poria (Baifuling) | MOL000282 | ergosta-7,22E-dien-3beta-ol | 43.51 | 0.91 |
|  | MOL000283 | ergosterol peroxide | 40.36 | 0.34 |
|  | MOL000300 | dehydroeburicoic acid | 44.17 | 0.18 |
| Saposhnikoviae Radix (Fangfeng) | MOL000011 | (2R,3R) …9-one | 68.83 | 0.66 |
|  | MOL011730 | 11-hydroxy-sec-o-beta-d-glucosylhamaudol_qt | 50.24 | 0.27 |
|  | MOL011732 | anomalin | 59.65 | 0.66 |
|  | MOL011737 | divaricatacid | 87.00 | 0.32 |
|  | MOL011749 | phelloptorin | 43.39 | 0.28 |
|  | MOL002644 | phellopterin | 40.19 | 0.28 |
|  | MOL001494 | mandenol | 42.00 | 0.19 |
|  | MOL001942 | isoimperatorin | 45.46 | 0.23 |

**Abbreviations:** 5-…furocoumarin: 5-[2'(R)-Hydroxy-3'-methyl-3'-butenyl-oxy]furocoumarin;

4-…7-one: 4-[(2S)-2,3-dihydroxy-3-methylbutoxy]furo[3,2-g]chromen-7-one;

(2R,3R,4S)…6-ol: (2R,3R,4S)-4-(4-hydroxy-3-methoxy-phenyl)-7-methoxy-2,3-dimethylol-tetralin-6-ol;

1S…1-carboxylate: [(1S)-3-[(E)-but-2-enyl]-2-methyl-4-oxo-1-cyclopent-2-enyl] (1R,3R)-3-[(E)-3-methoxy-2-methyl-3-oxoprop-1-enyl]-2,2-dimethylcyclopropane-1-carboxylate;

4a…acid: 4a-formyl-7alpha-hydroxy-1-methyl-8-methylidene-4aalpha,4bbeta-gibbane-1alpha,10beta-dicarboxylic acid;

(1R,2R)…enoate: [(1R,2R)-2,3-dihydroxy-1-(7-methoxy-2-oxochromen-6-yl)-3-methylbutyl](Z)-2-methylbut-2-enoate;

(2R,3R) …9-one: (2R,3R)-3-(4-hydroxy-3-methoxy-phenyl)-5-methoxy-2-methylol-2,3-dihydropyrano[5,6-h][1,4]benzodioxin-9-one;

(3S) …4-one: (3S)-7-hydroxy-3-(2,3,4-trimethoxyphenyl)chroman-4-one;

(2R) …4-one: (2R)-5,7-dihydroxy-2-(4-hydroxyphenyl)chroman-4-one;

1-…2,7-diol: 1-(4-hydroxybenzyl)-4-methoxy-9,10-dihydrophenanthrene-2,7-diol;

5,7…4-one: 5,7-dihydroxy-2-(3-hydroxy-4-methoxyphenyl)chroman-4-one;

(1R,2R)…methylbutanoate: [(1R,2R)-2,3-dihydroxy-1-(7-methoxy-2-oxochromen-6-yl)-3-methylbutyl] 3-methylbutanoate.

**Table S3** Target genes for Chinese herbs.

| Herb name | Target genes | | | | | |
| --- | --- | --- | --- | --- | --- | --- |
| Angelicae Dahuricae Radix (Baizhi) | GABRA3 | CASP3 | CTRB1 | MAP2 | PRKCA | BAX |
|  | ADH1C | CASP8 | DPP4 | NCOA1 | PTGS1 | BCL2 |
|  | ADRA1A | CASP9 | ESR1 | NCOA2 | PTGS2 | CHRM4 |
|  | ADRA1B | CCNA2 | GABRA1 | NR3C2 | RXRA | CHRNA2 |
|  | ADRA2A | CHEK1 | GABRA5 | OPRM1 | SCN5A | MAOA |
|  | ADRB1 | CHRM1 | JUN | PDE3A | SLC6A2 | MAOB |
|  | ADRB2 | CHRM2 | KCNH2 | PGR | SLC6A3 | PLAU |
|  | AR | CHRM3 | LTA4H | PIK3CG | SLC6A4 | PON1 |
| Ampelopsis Radix (Bailian) | GABRA3 | CHRM4 | FOS | MMP1 | PRKCB | CDKN1A |
|  | ACACA | CHRNA2 | GABRA1 | MMP2 | PRSS1 | CHEK1 |
|  | ACHE | CHUK | GABRA5 | MMP3 | PSMD3 | CHEK2 |
|  | ACP3 | CLDN4 | GJA1 | MMP9 | PTEN | CHRM1 |
|  | ADH1C | COL1A1 | GSK3B | MPO | PTGER3 | CHRM2 |
|  | ADRA1A | COL3A1 | GSTM1 | MYC | PTGS1 | ESR1 |
|  | ADRA1B | CRP | GSTM2 | NCF1 | PTGS2 | ESR2 |
|  | ADRA2A | CTRB1 | GSTP1 | NCOA1 | RAF1 | F3 |
|  | ADRB1 | CTSD | HAS2 | NCOA2 | RASA1 | F7 |
|  | ADRB2 | CXCL10 | HIF1A | NFE2L2 | RASSF1 | CHRM3 |
|  | AHR | CXCL11 | HK2 | NFKBIA | RB1 | MAOB |
|  | AHSA1 | CXCL2 | HMOX1 | NKX3-1 | RELA | MAP2 |
|  | AKT1 | CXCL8 | HSF1 | NOS3 | RUNX1T1 | MAPK1 |
|  | AR | CYP1A1 | HSPB1 | NPEPPS | RUNX2 | MAPK14 |
|  | BAX | CYP1A2 | ICAM1 | NQO1 | RXRA | MGAM |
|  | BCL2 | CYP1B1 | IFNG | NR1I2 | SCN5A | PPARA |
|  | BCL2L1 | CYP3A4 | IGF2 | NR1I3 | SELE | PPARD |
|  | BIRC5 | DCAF5 | IGFBP3 | NR3C2 | SERPINE1 | PPARG |
|  | CA2 | DIO1 | IL10 | ODC1 | SLC2A4 | PRKCA |
|  | CASP3 | DPP4 | IL1A | OPRM1 | SLC6A2 | TP53 |
|  | CASP8 | DUOX2 | IL1B | PARP1 | SLC6A3 | VCAM1 |
|  | CASP9 | E2F1 | IL2 | PCOLCE | SLC6A4 | VEGFA |
|  | CAT | E2F2 | IL6 | PDE3A | SOD1 | XDH |
|  | CAV1 | EGF | INSR | PGR | SPP1 | CD40LG |
|  | CCL2 | EGFR | IRF1 | PIK3CG | STAT1 | ERBB3 |
|  | CCNA2 | EIF6 | JUN | PLAT | THBD | MAOA |
|  | CCNB1 | ELK1 | KCNH2 | PLAU | TNF | POR |
|  | CCND1 | ERBB2 | LTA4H | PON1 | TOP1 | TOP2A |
| Typhonii Rhizoma (Baifuzi) | PTGS1 | PDE3A | BCL2 | PTGS1 | PDE3A | RXRA |
|  | PTGS2 | GABRA5 | BAX | PTGS2 | ADRB2 | NR3C1 |
|  | NCOA2 | ADRA1A | CASP9 | NCOA2 | ESR2 | GABRA1 |
|  | PGR | GABRA3 | JUN | NOS2 | DPP4 | NR3C2 |
|  | PIK3CG | CHRM2 | CASP3 | F2 | MAPK14 | KDR |
|  | KCNH2 | ADRA1B | CASP8 | KCNH2 | GSK3B | F7 |
|  | CHRM3 | ADRB2 | PRKCA | ESR1 | HSP90AA1 | PRKACA |
|  | CHRM1 | CHRNA2 | PON1 | AR | CHEK1 | PPARG |
|  | SCN5A | SLC6A4 | MAP2 | SCN5A | CCNA2 | NCOA1 |
|  | CHRM4 | OPRM1 | NR3C2 |  |  |  |
| Angelicae Sinensis Radix (Danggui) | PGR | CHRM3 | GABRA3 | BAX | NCOA1 | DRD1 |
|  | NCOA2 | CHRM1 | CHRM2 | CASP9 | ADRA2A | RXRA |
|  | PTGS1 | SCN5A | ADRA1B | JUN | SLC6A2 | BCL2 |
|  | PTGS2 | GABRA2 | ADRB2 | CASP3 | SLC6A3 | ADRB1 |
|  | HSP90AA1 | CHRM4 | CHRNA2 | CASP8 | PLAU | CHRNA7 |
|  | PIK3CG | PDE3A | SLC6A4 | PRKCA | LTA4H | NR3C2 |
|  | KCNH2 | GABRA5 | OPRM1 | PON1 | MAOB | CTRB1 |
|  | PRKACA | ADRA1A | GABRA1 | MAP2 | MAOA |  |
| Asari Radix et Rhizoma (Xixin) | PTGS1 | HTR3A | ACOX1 | AR | HAS2 | BAX |
|  | SCN5A | F7 | ACLY | PPARG | GSTP1 | TNF |
|  | PTGS2 | KDR | EHHADH | PGR | AHR | NR1I2 |
|  | NOS3 | CHRM4 | AUH | JUN | PSMD3 | NOX1 |
|  | RXRA | OPRD1 | HADHB | AHSA1 | SLC2A4 | KCNMA1 |
|  | PDE3A | CCND1 | CA2 | CASP3 | NR1I3 | PRSS1 |
|  | GABRA1 | IL10 | NCOA2 | MAPK8 | INSR | CCNA2 |
|  | PIK3CG | FASN | NOS2 | XDH | DIO1 | SELE |
|  | CHRM3 | ACACA | ESR1 | MMP1 | PPP3CA | VCAM1 |
|  | KCNH2 | G6PD | ACHE | STAT1 | GSTM1 | CHRM2 |
|  | CHRM1 | ECE1 | ADRA1B | HMOX1 | GSTM2 | RELA |
|  | CHRM5 | ACADM | ADRB2 | CYP3A4 | AKR1C3 | CYP1B1 |
|  | IKBKB | CYP2B6 | ADRA1D | CYP1A2 | SLPI | ICAM1 |
|  | AKT1 | SREBF1 | ESR2 | CYP1A1 | SLC6A2 | DPP4 |
|  | BCL2 | NOX3 |  |  |  |  |
| Pharbitidis Semen (Qianniuzi) | NR3C2 | ADRA1A | PON1 | RXRB | OPRD1 | SCN5A |
|  | PGR | GABRA3 | MAP2 | PRSS1 | SLC6A3 | CHRM4 |
|  | GABRA1 | CHRM2 | NOS2 | F7 | ADRA1D | CHEK1 |
|  | GABRA6 | ADRA1B | ESR1 | KDR | DRD2 | CASP8 |
|  | PTGS1 | ADRB2 | AR | CCNA2 | SLC6A2 | PRKCA |
|  | PTGS2 | CHRNA2 | PPARG | NCOA1 | ADRA2B | MAPK14 |
|  | PIK3CG | SLC6A4 | CHRM5 | KCNMA1 | ADRB1 | GSK3B |
|  | NCOA2 | OPRM1 | NOS3 | PYGM | ADRA2C | ADRA2A |
|  | JUN | BCL2 | RXRA | AMY2A | HTR2C | HTR3A |
|  | KCNH2 | BAX | ACHE | NOXA1 | DRD4 | PDE3A |
|  | CHRM3 | CASP9 | ESR2 | GABPB1 | HRH1 | GABRA5 |
|  | CHRM1 | CASP3 | DPP4 | DRD5 | HTR1B |  |
| Nardostachyos Radix et Rhizoma (Gansong) | PTGS1 | CHRM1 | CHRM2 | GABRA1 | TNF | NR3C1 |
|  | ESR1 | SCN5A | ADRA1B | RELA | EDN1 | OPRD1 |
|  | PTGS2 | CHRM5 | ADRB2 | STAT3 | BIRC5 | NCOA2 |
|  | PGR | CA2 | ADRA1D | CCND1 | CHRM3 | NCOA1 |
|  | NR3C2 | CHRM4 | OPRM1 | BCL2L1 | ADRA1A |  |
| Bletillae Rhizoma (Baiji) | NOS2 | ESR1 | PPARG | EGFR | MAPK14 | DPP4 |
|  | PTGS1 | AR | PTGS2 | ESR2 | GSK3B | RXRA |
|  | KCNH2 | SCN5A |  |  |  |  |
| Schizonepetae Herba (Jingjie) | PTGS2 | INSR | ODC1 | GSTP1 | CCNB1 | BCL2L1 |
|  | CA2 | CLDN4 | XDH | NFE2L2 | PLAT | FOS |
|  | PRSS1 | PPARA | CASP8 | NQO1 | THBD | CDKN1A |
|  | NCOA2 | PPARD | TOP1 | PARP1 | SERPINE1 | EIF6 |
|  | NCOA1 | HSF1 | RAF1 | AHR | COL1A1 | BAX |
|  | PTGS1 | CRP | SOD1 | PSMD3 | IFNG | CASP9 |
|  | SCN5A | CXCL10 | PRKCA | SLC2A4 | PTEN | NKX3-1 |
|  | PIK3CG | CHUK | MMP1 | COL3A1 | IL1A | RASA1 |
|  | AR | SPP1 | HIF1A | CXCL11 | MPO | PRXC1A |
|  | PPARG | RUNX2 | STAT1 | CXCL2 | TOP2A | GSTM1 |
|  | DPP4 | RASSF1 | RUNX1T1 | DCAF5 | NCF1 | GSTM2 |
|  | KCNH2 | E2F1 | ERBB2 | NR1I3 | HAS2 | PGR |
|  | ADRB2 | E2F2 | ACACA | CHEK2 | PLAU | VCAM1 |
|  | MMP3 | ACP3 | HMOX1 | NR3C2 | MMP2 | PTGER3 |
|  | F7 | CTSD | CYP3A4 | ADH1C | MMP9 | CXCL8 |
|  | RXRA | IGFBP3 | CYP1A2 | ADRA2A | MAPK1 | PRKCB |
|  | ACHE | IGF2 | CAV1 | SLC6A2 | IL10 | BIRC5 |
|  | GABRA1 | CD40LG | MYC | SLC6A3 | EGF | DUOX2 |
|  | MAOB | IRF1 | F3 | LTA4H | RB1 | GABRA3 |
|  | RELA | ERBB3 | GJA1 | MAOA | TNF | CHRM2 |
|  | EGFR | PON1 | CYP1A1 | CTRB1 | JUN | ADRA1B |
|  | AKT1 | DIO1 | ICAM1 | CHRM3 | IL6 | MGAM |
|  | VEGFA | PCOLCE | IL1B | CHRM1 | AHSA1 | IL2 |
|  | CCND1 | NPEPPS | CCL2 | ADRB1 | CASP3 | NR1I2 |
|  | BCL2 | HK2 | SELE | ADRA1A | TP53 | ADRA1B |
|  | CYP1B1 | HSPB1 | NOS3 | POR | ELK1 | NFKBIA |
| Notopterygii Rhizoma et Radix (Qianghuo) | KCNH2 | DPP4 | AR | ADRB2 | ADRA1B | CHRM1 |
|  | PTGS2 | NCOA1 | GABRA1 | SCN5A | ESR1 | RXRA |
|  | ACHE |  |  |  |  |  |
| Angelicae Pubescentis Radix (Duhuo) | PTGS2 | ESR1 | RXRA | OPRM1 | PDE3A | DPP4 |
|  | PTGS1 | AR | CHRM2 | GABRA1 | ACHE | ADRB2 |
|  | CHRM1 | SCN5A |  |  |  |  |
| Persicae Semen (Taoren) | GABRA3 | CASP3 | GABRA1 | NR3C2 | PTGS1 | BCL2 |
|  | ADH1C | CASP8 | GABRA5 | OPRM1 | PTGS2 | CA2 |
|  | ADRA1A | CASP9 | GABRA6 | PDE3A | RXRA | CHRM4 |
|  | ADRA1B | CHRM1 | GRIA2 | PGR | SCN5A | CHRNA2 |
|  | ADRB2 | CHRM2 | JUN | PIK3CG | SLC6A2 | MAP2 |
|  | BAX | CHRM3 | KCNH2 | PON1 | SLC6A3 | NCOA2 |
|  | PRSS1 | PRKCA | SLC6A4 |  |  |  |
| Scutellariae Radix (Huangqin) | ACHE | CASP3 | EGLN1 | KCNMA1 | PPARD | PLAU |
|  | ADRA1A | CASP8 | EIF6 | KDR | PPARG | PON1 |
|  | ADRA1B | CASP9 | ESR1 | LTA4H | PRKACA | KCNH2 |
|  | ADRA2A | CCNB1 | ESR2 | MAOA | PRKCA | PGR |
|  | ADRB1 | CCND1 | F10 | MAOB | PRKCD | DRD1 |
|  | ADRB2 | CDKN1A | F2 | MAP2 | PRSS1 | IL6 |
|  | AHR | CHEK1 | F7 | MAPK14 | PTGER3 | JUN |
|  | AHSA1 | CHRM1 | FASLG | MCL1 | PTGS1 | TP53 |
|  | AKT1 | CHRM2 | FASN | MMP1 | PTGS2 | HIF1A |
|  | APOD | CHRM3 | FN1 | MMP9 | PTPN1 | HSP90AA1 |
|  | AR | CHRM4 | FOS | MPO | PYGM | IGF2 |
|  | BAX | CHRNA2 | FOSL1 | NCOA1 | RELA | OPRM1 |
|  | BBC3 | CHRNA7 | FOSL2 | NCOA2 | RXRA | PDE10A |
|  | BCL2 | CTRB1 | GABRA1 | NFATC1 | SCN5A | PDE3A |
|  | CA2 | CXCL8 | GABRA2 | NOS2 | SLC6A2 | TEP1 |
|  | CACNA2D1 | CYCS | GABRA3 | NOS3 | SLC6A3 | TNF |
|  | PIK3CG | CYP2C9 | GABRA5 | NOX5 | SLC6A4 | TOP2A |
|  | PKIA | DPP4 | GSK3B | NR3C2 | TDRD7 |  |
| Poria (Baifuling) | PGR | AR | F7 | PTGS1 | ADRA1B | NOS3 |
|  | KCNH2 | PTGS2 | DPP4 | RXRA | GABRA1 | CHRM1 |
|  | ESR1 | NCOA2 |  |  |  |  |

**Table S4** Melanogenesis-related target genes for 10 candidate active ingredients.

| Active ingredients | Target genes | | | | |
| --- | --- | --- | --- | --- | --- |
| Quercetin | AKT1 | IL10 | SELE | SLC2A4 | PTGS1 |
|  | TNF | HMOX1 | PRKCA | BAX | COL3A1 |
|  | TP53 | CASP8 | CXCL10 | CYP3A4 | CYP1A2 |
|  | IL6 | ICAM1 | PARP1 | CYP1A1 | RASA1 |
|  | VEGFA | NOS3 | IGFBP3 | PRKCB | ADRB2 |
|  | JUN | RELA | AHR | GSTP1 | DUOX2 |
|  | IL1B | MAPK1 | RAF1 | CHEK2 | E2F2 |
|  | CASP3 | VCAM1 | CCNB1 | E2F1 | ACHE |
|  | EGFR | BCL2L1 | CHUK | CTSD | PPARD |
|  | PTGS2 | IFNG | CD40LG | BIRC5 | HSF1 |
|  | MYC | SERPINE1 | HSPB1 | PLAT | POR |
|  | HIF1A | IL2 | RUNX2 | CXCL11 | ODC1 |
|  | EGF | PPARA | PLAU | NR1I2 | RASSF1 |
|  | MMP9 | STAT1 | IGF2 | ERBB3 | NR1I3 |
|  | PPARG | NFE2L2 | F3 | TOP1 | XDH |
|  | HSP90AA1 | CASP9 | F2 | INSR | MGAM |
|  | CXCL8 | CAV1 | NQO1 | CYP1B1 | F7 |
|  | CCND1 | MMP3 | RB1 | BCL2 | PTGER3 |
|  | CCL2 | AR | COL1A1 | RXRA | ELK1 |
|  | PTEN | CRP | IRF1 | TOP2A | MAOB |
|  | FOS | SPP1 | CXCL2 | PRKACA | PRSS1 |
|  | ERBB2 | CDKN1A | SOD1 | PON1 | CLDN4 |
|  | MMP2 | MPO | NCF1 | GSTM1 | PCOLCE |
|  | NFKBIA | IL1A | DPP4 | HK2 | GABRA1 |
|  | EIF6 | MMP1 | GJA1 | PIK3CG |  |
| Kaempferol | TNF | RELA | PGR | BAX | XDH |
|  | AKT1 | AHR | CYP3A4 | NR1I2 | PPP3CA |
|  | PTGS2 | ICAM1 | GSTP1 | GSTM1 | INSR |
|  | JUN | NOS2 | CYP1B1 | PTGS1 | CHRM2 |
|  | CASP3 | VCAM1 | AR | CHRM1 | GABRA1 |
|  | PPARG | IKBKB | MMP1 | ACHE | SLPI |
|  | HMOX1 | STAT1 | SLC2A4 | BCL2 | PRSS1 |
|  | MAPK8 | SELE | DPP4 | PIK3CG | F7 |
|  | NOS3 | CYP1A1 | CYP1A2 | NR1I3 | SLC2A4 |
| Wogonin | AKT1 | RELA | GSK3B | PRKACA | PRSS1 |
|  | TP53 | CCND1 | AR | BBC3 | EIF6 |
|  | TNF | CDKN1A | KDR | PTGS1 | GABRA1 |
|  | CASP3 | CASP9 | NOS2 | DPP4 | ESR1 |
|  | IL6 | CXCL8 | BAX | RXRA | MAPK14 |
|  | HSP90AA1 | FN1 | MMP1 | PIK3CG | PRKCD |
|  | JUN | PPARG | CHEK1 | ADRB2 | TEP1 |
|  | PTGS2 | MCL1 | BCL2 | PTGER3 |  |
| beta-sitosterol | CASP3 | CASP8 | PGR | GABRA3 | ADRB2 |
|  | JUN | BAX | GABRA5 | MAP2 | MT-CO1 |
|  | HSP90AA1 | CHRM2 | DRD1 | PTGS1 | CHRM4 |
|  | PTGS2 | GABRA2 | BCL2 | OPRM1 | PON1 |
|  | CHRM1 | GABRA1 | PRKACA | PIK3CG | ADRA1A |
|  | CASP9 | CHRNA7 | PRKCA | CHRM3 |  |
| Baicalein | AKT1 | FOS | BAX | AHR | PRSS1 |
|  | TP53 | HSP90AA1 | IGF2 | PRKACA | TDRD7 |
|  | MMP9 | RELA | NFATC1 | DPP4 | APOD |
|  | PTGS2 | AR | MPO | EGLN1 | FOSL1 |
|  | HIF1A | CYCS | BCL2 | PTGS1 | PIK3CG |
|  | CASP3 | CCNB1 |  |  |  |
| Stigmasterol | SLC6A2 | ADRA2A | GABRA1 | PTGS1 | PRKACA |
|  | SLC6A3 | MAOA | PTGS2 | PLAU | RXRA |
|  | CHRNA7 | ADRB2 | ADRA1A | GABRA3 | MT-CO2 |
|  | CHRM2 | MAOB | ADRB1 | LTA4H | NR3C2 |
|  | CHRM1 | PGR | CHRM3 |  |  |
| Anhydroicariti | NOS3 | NOS2 | ESR2 | ACHE | CHEK1 |
|  | PPARG | AR | CHRM1 | RXRB | KCNMA1 |
|  | PTGS2 | GSK3B | RXRA | DPP4 | PRSS1 |
|  | ESR1 | KDR | CCNA2 | CHRM5 | F7 |
|  | MAPK14 | PTGS1 | ADRB2 | CHRM3 |  |
| AGROCLAVIN | SLC6A3 | PTGS1 | GABRA1 | ADRA1A | DRD4 |
|  | SLC6A2 | CHRM4 | CHRM2 | CHRM3 | HTR2C |
|  | PTGS2 | HTR3A | ADRB1 | CHRM1 | ADRA2C |
|  | OPRM1  HRH1 | CHRM5 | ADRA2A | ADRB2 | DRD2 |
| Moslosooflavone | HSP90AA1 | NOS2 | ESR2 | CHEK1 | DPP4 |
|  | PPARG | AR | PTGS1 | KCNMA1 | PRSS1 |
|  | PTGS2 | GSK3B | RXRA | F2 | GABRA1 |
|  | MAPK14  TDRD7 | PRKACA | PIK3CG | ADRB2 | CHRNA7 |
| Acacetin | TP53 | PTGS2 | BAX | BCL2 | PIK3CG |
|  | CASP3 | CASP8 | CHEK1 | PRKACA | DPP4 |
|  | HSP90AA1 | RELA | NOS2 | FASN | PRSS1 |
|  | CDKN1A | AR | FASLG | PTGS1 | ADRB2 |

(Genes are sorted from left to right according to their centrality)
